# Pharmaceutical Development of AAV-Based Gene Therapy Products for the Eye

**DOI:** 10.1007/s11095-018-2554-7

**Published:** 2018-12-27

**Authors:** Gerard A. Rodrigues, Evgenyi Shalaev, Thomas K. Karami, James Cunningham, Nigel K. H. Slater, Hongwen M. Rivers

**Affiliations:** 1 Biological Research, Allergan plc, Irvine, California 92612 USA; 2 Pharmaceutical Research and Development, Allergan plc, 2525 Dupont Drive, Irvine, California 92612-1531 USA; 3 Department of Chemical Engineering and Biotechnology, University of Cambridge, Cambridge, CB3 0AS UK

**Keywords:** adeno-associated virus (AAV) vector, formulation, gene therapy, ocular diseases, product development

## Abstract

A resurgence of interest and investment in the field of gene therapy, driven in large part by advances in viral vector technology, has recently culminated in United States Food and Drug Administration approval of the first gene therapy product targeting a disease caused by mutations in a single gene. This product, LUXTURNA™ (voretigene neparvovec-rzyl; Spark Therapeutics, Inc., Philadelphia, PA), delivers a normal copy of the *RPE65* gene to retinal cells for the treatment of biallelic *RPE65* mutation–associated retinal dystrophy, a blinding disease. Many additional gene therapy programs targeting both inherited retinal diseases and other ocular diseases are in development, owing to an improved understanding of the genetic basis of ocular disease and the unique properties of the ocular compartment that make it amenable to local gene therapy. Here we review the growing body of literature that describes both the design and development of ocular gene therapy products, with a particular emphasis on target and vector selection, and chemistry, manufacturing, and controls.

## Introduction

The term gene therapy refers to the treatment of human diseases using genetic methods, which can comprise either the introduction of a healthy copy of a flawed gene or correction of a gene to restore its biological function. The concept was initially proposed in the early 1990s (1), when both the knowledge of human genes and the understanding of molecular mechanisms underlying disease progression opened up the possibility of genetic intervention to achieve a therapeutic outcome. In the first wave of gene therapy, replication-defective retroviruses were utilized as gene delivery vectors, but the hope was soon dampened by concerns around side effects, including inflammatory responses to the vectors and the potential for genotoxicity caused by vector integration into the genome. In spite of the setbacks experienced in the early trials, advances in virology, immunology, and other related areas continued to provide improved vectors that promised to overcome these technical obstacles. In the 2000s, adeno-associated virus (AAV) vectors derived from non-pathogenic and non-enveloped replication-defective parvovirus emerged as a safe and efficient tool for gene delivery, triggering a second wave of clinical activity. Initial clinical gene therapy success was reported in 2008 by three independent groups that demonstrated the safety of subretinal injection of retinal pigment epithelium–specific 65-kDa protein (RPE65)–expressing AAV vector, leading to vision improvement in people with inherited blindness (2–4). As a result, a comeback for gene therapy was declared (5). Eventually, in 2017, LUXTURNA™ (voretigene neparvovec-rzyl) from Spark Therapeutics, Inc. (Philadelphia, PA) became the first United States Food and Drug Administration (FDA)–approved gene therapy product for the eye.

Although gene therapy is being pursued as a strategy for the treatment of a range of genetic diseases, ocular disorders are particularly attractive targets for this type of therapy. Individual genes responsible for a range of ocular disease conditions, including inherited blindness, have been identified. In the posterior segment of the eye, retinal cells are post-mitotic, allowing for sustained gene expression without the need for genomic integration of the transgene. The eye has a well-defined anatomy with a limited and closed physical space that offers unique advantages for local delivery, including the ability to directly visualize and access the tissue. In addition, the blood-ocular barrier contributes to ocular immune privilege and limits immunologic responses to gene therapy products. Gene therapy also offers the potential for long-lasting treatment, mitigating the treatment burden often associated with local delivery to the posterior segment (e.g., intravitreal injection of anti–vascular endothelial growth factor [VEGF] biologics). Finally, the significant progress of ophthalmology research, including the availability of relevant animal models, also contributes to the intensified activities in ocular gene therapy.

Inherited retinopathies such as Leber congenital amaurosis type 2 (LCA2), which is associated with mutations in the *RPE65* gene, have been the focus of early clinical interest and success. By 2013, persistent visual improvement as well as safety were reported in clinical studies for up to 3 years following a single subretinal injection of AAV vectors expressing *RPE65* in LCA2 patients (6–10). The safety, including immune tolerability, and efficacy of a second injection in the contralateral eye were further demonstrated by Bennett *et al*. (11).

Multiyear follow-up evaluation of the patients from two other trials (ClinicalTrials.gov NCT00481546 and NCT00643747), however, revealed progressive decline of clinical benefits including retinal sensitivity, visual acuity, and functional gain following an initial peak seen at 6–12 months after the treatment (12,13). This result underscores the challenge of using gene therapy as a long-term treatment. The FDA approval of sequential and bilateral injection of voretigene neparvovec-rzyl to treat visually impaired patients who carry an *RPE65* mutation was based on 1-year data from the only randomized controlled phase III clinical study to date, which demonstrated significant vision improvement as a result of the treatment (14).

With the clinical success for LCA2 patients, vector-based gene transfer is now being explored clinically for other forms of hereditary retinal diseases, including choroideremia (15), Leber hereditary optic neuropathy (LHON; ClinicalTrials.gov NCT01267422 and NCT02161380), Stargardt disease (ClinicalTrials.gov NCT01367444), X-linked retinoschisis (XLRS), and X-linked retinitis pigmentosa (XLRP) (Table I). In addition to monogenic inherited retinal disorders, gene therapy is also being explored to treat various corneal diseases associated with inherited mutations. These efforts are currently mostly limited to animal studies (16).

**Table I:** Summary of Active Ocular Gene Therapy Programs

Company	Program/ Product	Disease/ Mechanism	Vector Technology	Administration Route	Status	References
Spark Therapeutics, Inc.	LUXTURNA™ (voretigene neparvovec-rzyl)	Confirmed biallelic RPE65 mutation–associated retinal dystrophy; *RPE65* gene delivery	AAV2	Subretinal injection	US approval (2017)	Prescribing information
Nightstar Therapeutics	NSR-REP1	Choroideremia; *REP1* gene delivery	AAV2	Subretinal injection	Phase III	Company website NCT03496012
	NSR-RPGR	X-linked retinitis pigmentosa; codon-optimized *RPGR* gene delivery	AAV	Subretinal injection	Phase I/II	Company website NCT03116113
	NSR-BEST1	Best vitelliform macular dystrophy; *BEST1* gene delivery	Undisclosed	Undisclosed	Preclinical	Company website
RegenXBio Inc.	RGX-314	Wet AMD; anti-VEGF monoclonal antibody fragment	NAV AAV8	Subretinal injection	Phase I	Company website
Applied Genetic Technologies Corporation	XLRS (with Biogen, Inc.)	X-linked retinoschisis; *hRS1* gene delivery	rAAV2tYF	Intravitreal injection	Phase I/II	April 10, 2018 press release
	ACHM B3	Achromatopsia; *hCNGB3* gene delivery	rAAV2tYF	Subretinal injection	Phase I/II	Company website NCT02599922
	ACHM A3 / AGTC-402	Achromatopsia; *hCNGA3* gene delivery	rAAV2tYF	Subretinal injection	Phase I/II	Company website NCT02935517
	XLRP (with Biogen, Inc.)	X-linked retinitis pigmentosa; *RPGR* gene delivery	rAAV2tYF	Subretinal injection	Phase I/II	April 18, 2018 press release
GenSight Biologics	GS010 (rAAV2/2-ND4)	LHON	AAV2	Intravitreal injection	Phase I/II	NCT02064569
National Eye Institute, US National Institutes of Health	scAAV2-P1ND4v2	LHON	AAV2	Intravitreal injection	Phase I	NCT02161380
Sanofi Genzyme	SAR422459 (with Oxford BioMedica)	Stargardt disease; *ABCR* gene delivery	Lentivirus (LentiVector)	Subretinal injection	Phase II	Company website
	SAR421869 (with Oxford BioMedica)	Usher syndrome type 1B; *MYO7A* gene delivery	Lentivirus (LentiVector)		Phase I/II	Company website
Allergan plc	RST-001 (acquired RetroSense Therapeutics LLC)	Retinitis pigmentosa; channelrhodopsin-2 optogenetic gene therapy	Undisclosed	Intravitreal injection	Phase I/II	Press releases NCT02556736
Oxford BioMedica	OXB-201	Wet AMD; endostatin and angiostatin gene delivery	Lentivirus (LentiVector)	Subretinal injection	Phase I	Company website
	OXB-202	Corneal graft rejection; endostatin and angiostatin gene delivery	Lentivirus (LentiVector)	Treatment of cornea prior to transplantation	Preclinical	Company website
National Eye Institute, US National Institutes of Health	RS1 AAV vector	XLRS; *RS1* gene delivery	AAV8	Intravitreal injection	Phase I/IIa	NCT02317887
Eyevensys	EYS606	Noninfectious uveitis; anti–tumor necrosis factor-α plasmid delivery	EyeCET (electrotrans-fection)	Ciliary muscle transfection	Phase I/II	Company website
	EYS609	Retinal vein occlusion/diabetic macular edema/wet AMD; anti-VEGF plasmid delivery	EyeCET (electrotrans-fection)	Ciliary muscle transfection	Preclinical	Company website
	EYS611	Retinal degeneration; neurotrophic factor plasmid delivery	EyeCET (electrotrans-fection)	Ciliary muscle transfection	Preclinical	Company website
Adverum Biotechnologies (formerly Avalanche Biotherapeutics)	ADVM-032	Wet AMD; anti-VEGF (ranibizumab)	AAV.7 m8 (4DMT)	Intravitreal injection	Undisclosed	
	ADVM-022	Wet AMD; anti-VEGF (aflibercept)	AAV.7 m8 (4DMT)	Intravitreal injection	Preclinical	Company website
	AVA-311 (with Regeneron)	XLRS; *RS1* gene delivery	Undisclosed	Intravitreal injection	Research	
4D Molecular Therapeutics	4D-110 (with Roche)	Choroideremia; REP-1	Therapeutic vector evolution	Intravitreal injection	Preclinical	Company website
	4D-125	Undisclosed	Therapeutic vector evolution	Intravitreal injection	Preclinical	Company website
Eos Neuroscience	Eos-013	Optogenetic gene therapy	AAV		Preclinical	Company website
Benitec Biopharma	BB-201	Wet AMD	Novel AAV	Intravitreal injection	Preclinical	Company website
iVeena	IVMED-50	Wet AMD; *Flt23k* gene delivery	AAV	Intraocular injection	Preclinical	Company website

Gene therapy can also be a powerful approach for treating non-hereditary chronic conditions such as age-related macular degeneration (AMD) and diabetic retinopathy. In particular, for AMD, current anti-VEGF treatments including Lucentis® (ranibizumab; Genentech, Inc., South San Francisco, CA) and Eylea® (aflibercept; Regeneron Pharmaceuticals, Inc., Tarrytown, NY) require frequent intravitreal injections and, as a result, present significant risks for patient compliance and therapeutic outcomes. Intensive efforts are under way to prolong treatment intervals, but the success with conventional sustained-release formulations or devices has thus far been limited. With the advancement of gene therapy technologies, multiple efforts are now being pursued at both the preclinical and clinical stages to achieve sustained expression of a VEGF-neutralizing protein in the posterior segment of the eye, promising a prolonged therapeutic effect from a single injection (Table I).

As gene therapy is explored as a treatment modality for an increasing number of ocular disease conditions, pharmaceutical development of gene therapy products is also progressing rapidly to help establish a powerful new class of therapeutic products. AAVs have emerged as the predominant vectors for delivering genes of interest to target tissues with improved specificity, efficiency, and safety. Development of these complex products, which consist of both viral genome and multiple structural proteins, faces numerous technical challenges. Formulation and production of AAV products requires carefully selected conditions to ensure good stability and yield, as some of the common processing methods employed for biologics, such as filtration or lyophilization, may lead to aggregation or loss of AAV titer. Maintaining stability is also a major challenge when choosing storage conditions. Also, because of the inherent complexity of AAV products, an array of advanced analytical tools is often required to provide sufficient understanding of the physiochemical properties, purity, and potency of the drug substance (DS) and product (DP). In parallel with these technical advancements, regulatory guidelines are also being adapted to clarify regulatory expectations for gene therapy products.

Overall, gene therapy has emerged as a transformational platform that can provide new treatment options for numerous ocular diseases, and its potential is only beginning to be realized. Although evolving fast, gene therapy is still at the early stage of its development as a new therapeutic modality. It is therefore our intent to provide a review on the current state of the technology from the perspectives of both product design and pharmaceutical development. Gene editing products, such as those involving the use of CRISPR-Cas9 or transcription activator-like effector nucleases (TALENs), are not covered in this review.

## Ocular Application of Gene Therapy: Diseases, Delivery and Vector Design

### Ocular Diseases

Over the last two decades, the underlying genetic factors contributing to disease pathology have been identified for a range of ocular diseases and have provided an opportunity to exploit the emerging field of gene therapy as a therapeutic approach. In particular, understanding of the genetics of inherited retinal diseases (IRDs) has grown dramatically (17). Not only have disease-causing genes been identified for IRDs but specific variants associated with pathology have been mapped. These advances have not been limited to monogenetic ocular disorders, as a number of gene variants have been identified that are associated with susceptibility or resistance to polygenic diseases such as AMD (18). Nor have they been limited to retinal diseases. For example, in glaucoma, many of the underlying genetic causes of hereditary forms of the disease have been identified (19), and causal genetic mutations have been identified in multiple genes associated with Fuchs corneal dystrophy (20).

#### Inherited Retinal Diseases

Genetic linkage analysis led to the identification of the first IRD genes, and the pace of discovery of disease genes and variants accelerated with the advent of DNA sequencing techniques including, most recently, next-generation sequencing (17,21). Currently, at least 260 genes associated with these disorders have been recognized, with many others remaining to be documented (22). Identification of these genes and their variants has also allowed the development of animal models of disease, which has not only increased our understanding of disease mechanism, but has also provided preclinical models to assess potential therapeutics, including gene therapy approaches (23). Prior to the emergence of molecular genetics, IRDs were classified largely on the basis of clinical presentation, region of the retina affected, disease progression, and inheritance patterns. Although a unified classification system has not yet been established for IRDs, a clinical classification system was recently devised that includes higher-order grouping of IRDs based on similar genetic causes (24). These categories are composed of photoreceptor diseases, macular diseases, and third branch disorders. The photoreceptor disease category includes diseases characterized by degeneration of rods and cones, and that can be broadly classified as either isolated or syndromic. The isolated diseases can be further subclassified based on whether they are acquired and progressive, as in the case of retinitis pigmentosa (RP) and cone and rod dystrophies, or congenital and stationary, as in the case of LCA or achromatopsia. Syndromic diseases include disorders affecting multiple organs such as Usher syndrome, which is characterized by hearing loss in addition to vision defects. RP is the most common subgroup of IRDs affecting photoreceptors and the retinal pigment epithelium (RPE), with an incidence of one in 3000–7000 individuals (25). Mutations in more than 70 genes have been associated with RP (22). The inherited macular dystrophies include disorders such as Stargardt disease and Best disease, which are characterized by central visual loss and atrophy of the macula and underlying RPE. The third branch disorders include choroidopathies such as choroideremia, retinoschisis such as XLRS, optic neuropathies such as LHON, and vitreoretinopathies such as Norrie disease (24).

Across all categories of IRDs, the patterns of inheritance include autosomal recessive, autosomal dominant, and X-linked. Additionally, less common mitochondrial forms of retinal disease have also been identified. As gene therapy is essentially a gene augmentation or replacement therapy, and recessive and X-linked null mutations result in the absence of a functional protein, these types of genetic alterations are most amenable to gene therapy approaches. This fact is reflected in the choice of targets for most of the ongoing gene therapy clinical trials, which include trials of gene therapy for the autosomal recessive IRDs Stargardt disease and achromotopsia, X-linked diseases including XLRS, choroideremia, and X-linked RP, as well as the mitochondrial inherited disease LHON (Table I). Interestingly, Nightstar Therapeutics (London, UK) has a preclinical program for *BEST1* in Best vitelliform macular dystrophy, an autosomal dominant disease.

#### Age-Related Macular Degeneration

AMD is a complex multifactorial condition with both genetic and environmental factors contributing to disease pathogenesis. Genome-wide association studies have led to the identification of single-nucleotide polymorphisms (SNPs) associated with increased or decreased incidence of the disease (26). Many of the SNPs occur in genes encoding components of the complement pathway (27). However, the potential to exploit this increased understanding of AMD genetics in order to develop gene therapies has yet to be explored clinically. One approach that is currently being pursued is to target factors not necessarily genetically linked to, but known to be involved in, disease pathogenesis. In the case of neovascular AMD, the role of VEGF as a principal mediator of neovascularization is well established, a fact underscored by the success of anti-VEGF therapies in treating the disease (28). Despite the availability of pharmacological treatments, gene therapy is an attractive approach because it offers the potential for a prolonged treatment effect, obviating the need for monthly intraocular injections. Consequently, a number of anti-VEGF gene therapy trials for AMD have been completed or are ongoing (Table I). Both Adverum Biotechnologies (Menlo Park, CA) and Sanofi Genzyme (Framingham, MA) completed phase I/II studies of AAV vectors expressing soluble fms-like tyrosine kinase-1 (sFlt-1), the soluble extracellular domain of VEGF receptor-1, which acts as a trap for VEGF. Both studies demonstrated the safety of AAV2-sFlt delivered either intravitreally or subretinally but showed limited efficacy (29,30). Adverum Biotechnologies has since shifted focus and is now employing a next-generation AAV2 vector (AAV.7 m8) administered intravitreally to express the anti-VEGF molecules ranibizumab and aflibercept, the latter of which they plan on advancing into clinical trials. Similarly, RegenXBio Inc. (Rockville, MD) is currently conducting a phase I trial using an AAV8 vector delivered subretinally to express ranibizumab (Table I). Interestingly, iVeena (Salt Lake City, UT) has a preclinical stage anti-VEGF gene therapy, IVMED-50, an AAV vector that expresses Flt23k. This molecule consists of the VEGF-binding domains 2–3 of Flt-1 coupled to KDEL, a tetrapeptide that binds endoplasmic reticulum retention receptors. Flt23k binds intracellular VEGF and sequesters it in the endoplasmic reticulum, where it is eventually degraded. In addition to the anti-VEGF gene therapy strategies, other antiangiogenic targets currently are being explored for gene therapy, including a lentivirus expressing endostatin and angiostatin that is currently in phase I evaluation (Table I).

### Routes of Administration

The success of gene therapy depends on efficient delivery of the genetic material to the target cells. Most gene defects associated with IRDs affect the development or function of photoreceptors and, to a lesser extent, cells of the RPE. Delivery of genes to these and other retinal cell types presents a challenge because there are physical barriers that need to be overcome regardless of the route of administration. In principle, ocular gene delivery can be performed via multiple routes including topical instillation, periocular routes, intracameral injection, intravitreal injection, subretinal injection, or suprachoroidal injection. In practice, the target cell type and the delivery system dictate the route of administration. Topical instillation, the least invasive route of administration, is also the least efficacious for non-viral delivery, as bioavailability of nucleic acids is generally low and penetration into the cornea and conjunctival epithelium is inefficient, limiting the utility of this approach for anterior segment diseases (31). Similarly, periocular routes such as retrobulbar, subtenon, or subconjunctival injections have limited utility for non-viral delivery due to the inability of large molecular weight nucleic acids to penetrate ocular tissue (31). However, viral delivery does not have these same limitations, as subconjunctival injections of AAV expressing antiangiogenics have shown efficacy in inhibiting corneal neovascularization in animal models (32,33). The intracameral space is another potential route to target anterior segment tissues with nucleic acids, albeit an inefficient one (31). In contrast, adenovirus delivery into the intracameral space has been used to successfully deliver a metalloproteinase gene into cells of the trabecular meshwork to control intraocular pressure in an animal model (34).

Intravitreal and subretinal injections are the two most common routes of administration for viral-based gene therapy for retinal diseases. Intravitreal injection is an accepted and relatively safe route of administration. It is the preferred route for targeting the retinal ganglion cells for the treatment of diseases like LHON but can also be used to target photoreceptors and the RPE. Although intravitreal delivery appears to be a direct path to the retina, there are anatomical barriers that prevent diffusion of viruses into the retina, most notably the inner limiting membrane (35). Strategies to enhance transduction of AAV following intravitreal administration include mild enzymatic digestion or surgical peeling of the inner limiting membrane (35,36). Additionally, second-generation AAV vectors are being designed to overcome these anatomical barriers (see the discussion of AAV vectors below). In contrast to intravitreal administration, subretinal injection of AAV is more invasive, with a risk of damage to the retinal tissue. Potential complications from the procedure include macular holes, subretinal hemorrhage, subretinal fibrosis, and retinal detachment. However, subretinal administration provides the most direct access to photoreceptors and the RPE. Although the spread of virus beyond the bleb formed at the injection site is limited (37), in most cases this is acceptable if the bleb is in the macular region where rescue or maintenance of visual function is most critical. In fact, the first approved ocular gene therapy, voretigene neparvovec-rzyl, is administered subretinally to treat LCA2 (14). Suprachoroidal administration involves delivery into a space between the sclera and the choroid and offers a less invasive alternative to subretinal injections. Although experience with this route of administration is limited, one animal study report has demonstrated the potential safety and efficacy of suprachoroidal delivery of AAV to the retina (38).

### Vector Considerations for Gene Delivery

Several different non-viral and viral-based vector systems have been employed for gene therapy, including liposomes and nanoparticles, AAV, lentivirus, and adenovirus. A comparison of the characteristics of the various delivery systems for gene therapy is presented in Table II.

**Table II:** Summary of Gene Delivery Systems: General Characteristics, Benefits and Limitations

Gene Delivery Vectors	Characteristics	Benefits	Limitations
Adeno-associated Viral Vectors	Single-stranded DNA genome Derived from replication-defective parvovirus Vector DNA remains episomal in cells 13 Serotypes identified in primates	Non-pathogenic, nonintegrating vectors Long-term transgene expression achievable	Cannot package more than ~4.5 kb of transgene DNA Long-term expression limited to post-mitotic cells High incidence of pre-existing immunity to AAV in humans
Lentivirus Vectors	Single-stranded RNA genome Vector DNA integrates into genome	Can accommodate transgenes up to 10 kb Genomic integration allows for sustained transgene expression in dividing cells Low immunogenicity	Low production yields Semi-random genomic integration pattern increases risk for insertional mutagenesis
Adenoviral Vectors	Double-stranded DNA genome Vector DNA remains episomal in cells More than 50 human serotypes identified; most utilized for gene therapy is Ad5	Large transgene capacity up to 37 kb High transduction efficiency of both dividing and non-dividing cells	Can elicit strong antiviral immune response Long-term expression limited to post-mitotic cells
Non-viral Gene Delivery	Comprises a variety of approaches including liposome and polymer-based nanoparticle carriers as well as physical methods like electroporation and iontophoresis	Lower risk for immunogenicity and insertional mutagenesis compared with the viral vectors^.^	Lower transfection efficiency than viral vectors Lack of persistent gene expression in cells limits duration of effect

#### Non-viral Delivery Systems

Non-viral approaches are currently being explored for ocular delivery as they offer some advantages over viral vectors, including a potentially more favorable safety profile because of a lower risk for immunogenicity or insertional mutagenesis, as well as the ability to deliver large DNA fragments. Here we highlight a few key approaches currently being explored. For more extensive reviews of non-viral vectors for gene therapy see Bloquel *et al*. (31) and Ramamoorth and Narvekar (39). The most notable strategies include lipid-based and polymer-based carriers. The challenge for non-viral delivery remains the requirement to overcome physical barriers present in the eye. Liposomes comprise amphiphilic molecules such as phospholipids and cholesterol. Inclusion of positively charged or titratable components can promote complexation with negatively charged DNA. As cellular membranes are composed of a phospholipid bilayer, liposomes can be designed to fuse with and overcome the cell membrane barrier. Indeed, liposomes can mediate uptake into RPE cells following subretinal injection (40). Solid lipid nanoparticles composed of an aqueous dispersion of a layer of surfactants surrounding a solid lipid core, and ranging in size from 50 to 1000 nm (16), are another vehicle that is being explored. Subretinal injections of solid lipid nanoparticles have resulted in transfection of RPE cells *in vivo* (41). Bioerodible polymers such as polyethyleneimine, polyesters like poly(lactic acid) (PLA) and poly(lactic-*co*-glycolic acid) (PLGA), chitosan, and hyaluronic acid have also been investigated for gene therapy (16). However, polyethyleneimine-based polymers have not shown efficient delivery to the retina following intravitreal injection (42). In contrast, internalization of PLA and PLGA nanoparticles by RPE cells *in vitro* has been demonstrated (43). Additionally, *in vivo* experiments have established that PLA nanoparticles are well tolerated and can cross the retina following intravitreal administration, with a preferential localization to RPE cells (44). Electro-transfection is an additional non-viral approach that has entered clinical development. Eyevensys (Paris, France) has developed the EyeCET platform, which involves injection of plasmid DNA into the ciliary muscle in conjunction with electrotransfection to enable production of therapeutic proteins *in vivo*.

#### Lentivirus

Lentiviruses are single-stranded RNA vectors that can infect both dividing and non-dividing cells. Because lentiviruses integrate their DNA into the chromosome of target cells, they can mediate sustained transgene expression even in dividing cells. Lentiviruses can overcome a major limitation of AAV vectors: the limited packaging capacity of ~4.8 kilobase (kb), with the practical limit often lower because of the need to include appropriate regulatory elements in the transgene cassette. In contrast, lentiviruses can accommodate transgenes in the range of 9–10 kb (45). Lentiviruses can effectively transduce RPE cells but not photoreceptors (46,47). Although insertional mutagenesis remains a concern with lentivirus, several approaches are being utilized to address this liability. For example, inactivation of the 3′ long terminal repeat generates self-inactivating vectors (48). Additionally, non-integrating vectors have been developed and shown to facilitate efficient sustained transgene expression in RPE for 8 weeks in a rodent model (49). Though less popular than AAV, clinical trials employing the use of lentivirus-mediated gene delivery are ongoing (Table I).

#### Adenovirus

Adenoviruses are double-stranded DNA vectors that can efficiently transduce both dividing and non-dividing cells. Adenovirus offers advantages over other viral vectors, including the ability to package large payloads of up to 37 kb (45). Moreover, as adenovirus remains episomal, there is minimal risk for insertional mutagenesis (45). There are more than 50 different adenovirus serotypes with different tropisms. Serotypes Ad5 and Ad2 vectors can transduce RPE and, to a lesser extent, photoreceptors (45). Ad5 serves as the basis for many current adenovirus gene therapy vectors. Both the tyrosine-protein kinase Mer and RPE65 have been expressed in RPE using Ad5 delivered subretinally and were shown to rescue function in animal models (50,51). Additionally, data from a phase I clinical trial using Ad5 intravitreally to deliver pigment epithelium-derived factor for neovascular AMD demonstrated safety and some evidence of a sustained therapeutic effect (52). One potential downside of the use of adenovirus is that it can induce immune responses that can limit transgene expression, even in the subretinal space (45). Cytotoxic T-cells have been shown to remove transduced cells expressing adenovirus proteins (53). Newer helper-dependent adenovirus vectors containing only the inverted terminal repeats and the packaging recognition signal, along with the transgene, have been developed that allow for long-term transgene expression (54).

#### Adeno-Associated Virus Vectors

AAV has emerged as the predominant vector for ocular gene therapy (6). AAV is a small (25-nm), non-enveloped virus belonging to the Parvoviridae family that offers a number of advantages as a delivery system, including favorable retinal cell transduction properties (55). Additionally, because AAV requires a helper virus for replication, it is generally considered to be non-pathogenic and, once a cell is transduced, it remains episomal, reducing the potential for genomic integration events. There are 12 different AAV serotypes that have been identified in primates to date, many of which are commonly used in gene therapy studies. The various AAV serotypes differ in their capsid components and thus display differential cellular tropism, transduction efficiency and immunogenicity. AAV1, 2, 4, 5, 6, 7, 8 and 9 all display tropism for retinal tissue (56–58). However, whereas all of these serotypes efficiently transduce RPE, transduction of photoreceptors is variable (45).

**Fig. 1:**
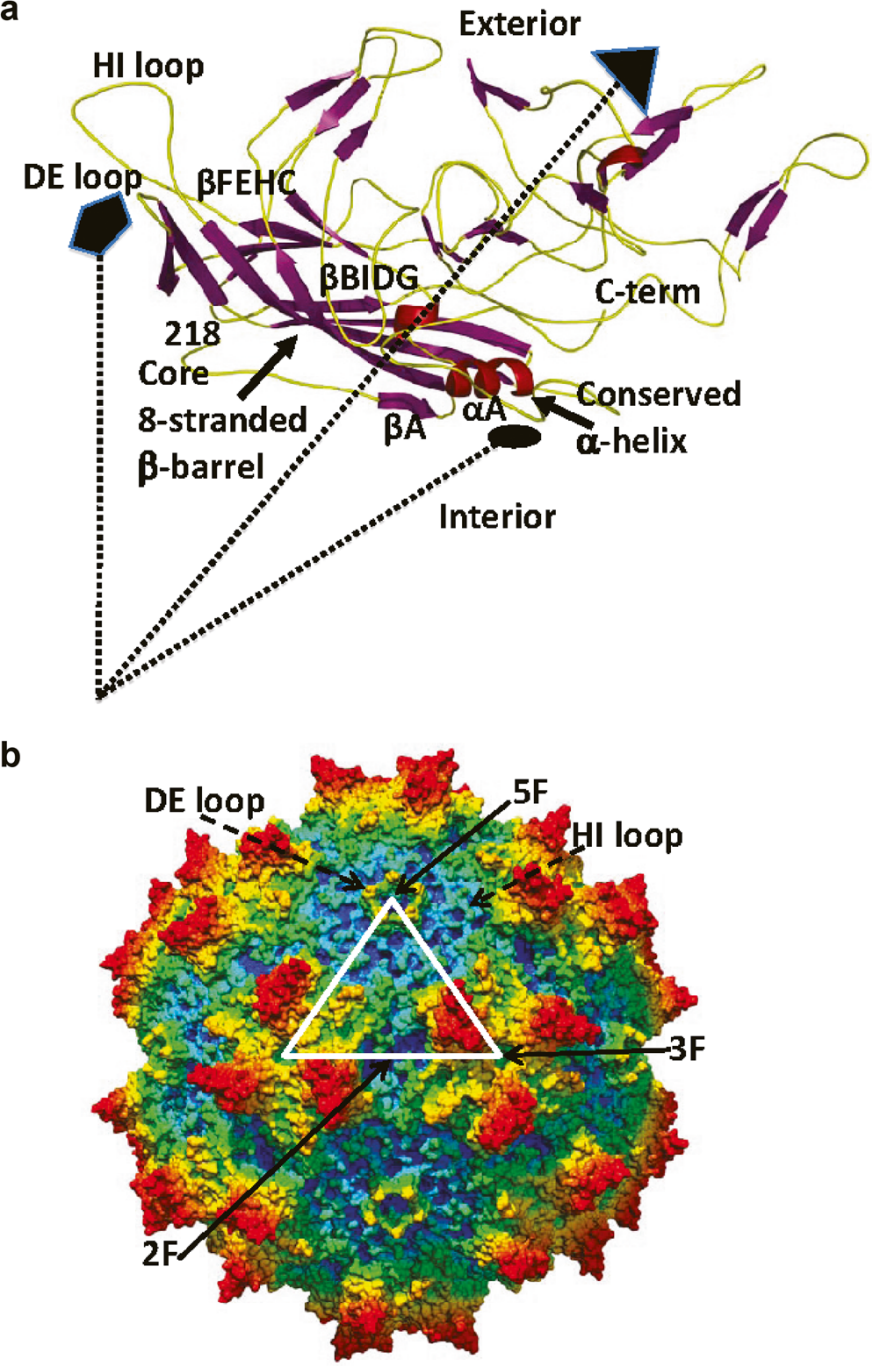
Adeno-associated virus serotype 1 (AAV1) structure. (**a**) Crystal structure of AAV1 capsid VP3 monomer (PDB ID, 3NG9). The β-strands are shown in purple ribbon, the conserved α-helix A is in red, and loops between the strands are in yellow. The dotted lines show the relative positions of the 5-fold (filled pentagon), 3-fold (filled triangle), and 2-fold (filled oval) interfaces of symmetry from the center of the capsid. An eight-stranded β-barrel (with β-sheets βCHEF and βBIDG), along with βA (labeled) and α-helix A (αA), forms the core of the VP monomer structure, flanked by large loop regions. The DE and HI loops (between β-strands D and E and between H and I, respectively) as well as the first ordered N-terminal residue (218), the C-terminus, and the interior and exterior capsid surfaces are labeled. (**b**) Radially color-cued (from capsid center to surface, blue to green to yellow to red) surface representation of the AAV1 capsid. The white triangle depicts a viral asymmetric unit bounded by one 5-fold axis and two 3-fold axes with a 2-fold axis between them. The approximate locations of the icosahedral 2-fold (2F), 3F, and 5F axes are indicated by the black arrows. The positions of the DE and HI loops are indicated by the dashed arrows. *Reproduced from Venkatakrishnan et al*. *J Virol. 2013;87:4974–84. doi* 10.1128/JVI.02524-12 (102) *with permission from American Society for Microbiology. © 2013.*

The AAV genome is packaged within an icosahedral capsid containing three open reading frames (ORFs) flanked by inverted terminal repeats. The rep ORF encodes for four proteins (Rep40, Rep52, Rep68, and Rep78) required for viral replication, transcriptional regulation, genome integration, and virion assembly. The cap ORF encodes three structural proteins, VP1, VP2, and VP3, which comprise the 60-subunit AAV capsid and assemble in icosahedral geometry to encapsulate the viral genome (Fig. 1). The three capsid proteins contain a common β-barrel domain but different N-terminal extensions (59). Variable loops create specific surface topologies for each AAV variant that mediate the molecular interactions responsible for cell association, entry, and immunological properties. Following initial binding to cell surface glycans (e.g., heparin sulfate, N-linked sialic acids, galactose, etc.), the entry of AAV into target cells is mediated by interactions with co-receptors such as fibroblast growth factor receptor, epidermal growth factor receptor, and platelet-derived growth factor receptor. Recombinant AAV (rAAV) vectors have been designed for gene therapy by replacing the rep and cap genes with the transgene cassette. The rep and cap ORFs are then provided in trans during AAV production using helper plasmids. rAAV2, the first serotype to be successfully used for gene transfer, efficiently transduces RPE and retinal ganglion cells but is less efficient in transducing photoreceptors (47,60,61). Importantly, capsid proteins can be exchanged among various AAV serotypes. Transient transfection of the different vector components in trans allows for exchange of capsids between different serotypes resulting in hybrid or “pseudotyped” AAV vectors (62). For example, many recombinant vectors in use today are composed of components of the AAV2 serotype combined with the capsids of AAV1 (AAV2/1), AAV4 (AAV2/4), AAV5 (AAV2/5), AAV6 (AAV2/6), AAV7 (AAV2/7), AAV8 (AAV2/8) and AAV9 (AAV2/9). Apart from helping to achieve the desired tropism and enhancing transduction efficiency, pseudotyping can also help circumvent pre-existing immunity to certain AAV serotypes that could affect the efficacy and potentially the safety of a gene therapy. For example, depending on geographic region, 30–60% of humans have neutralizing antibodies to AAV2, which have the potential to inhibit cell transduction (63). All AAV pseudotypes transduce RPE, with AAV2/1, AAV2/4, and AAV2/6 being the most specific (45). In contrast, only AAV2/5, AAV2/7, AAV2/8, and AAV2/9 efficiently transduce photoreceptors, with AAV2/8 and AAV2/9 being most efficient across a number of species (47,56,61,64–67). AAV2/5, AAV2/8, and AAV2/9 transduce the highest percentage of cone photoreceptors (61,66,68–70).

Recently, the array of AAV vectors has been expanded beyond naturally occurring serotypes as second-generation vectors have been designed or isolated that have greater transduction efficiency or altered tropism. Two different approaches have been used to generate these new vectors: rational design and directed evolution. Rational design leverages knowledge of structure/function relationships to modify the virus capsid structure, for example by introducing mutations to reduce proteasomal degradation or to eliminate antibody-binding epitopes, or by incorporating new ligands as a means to redirect vector tropism (71). Variants have been generated by site-directed mutagenesis of surface-exposed tyrosine residues on AAV2, which prevents capsid phosphorylation and subsequent ubiquitination and proteasome-mediated degradation (72). This in turn leads to enhanced nuclear transport and increased transgene expression (72). AAV2, AAV8, and AAV9 carrying these mutations have been shown to have increased transduction efficiency both *in vitro* and *in vivo* (72,73). Additionally, these mutations have the potential to reduce major histocompatibility complex class 1 presentation of capsid antigens because of reduced proteasomal degradation of proteins (74).

The directed evolution approach assumes no prior knowledge of structure/function relationships, but rather relies on the introduction of random genetic diversity, in conjunction with selection pressure, to accumulate mutations that achieve the targeted improvement in a process mimicking natural evolution. AAV libraries are generated by random mutagenesis of the capsids and then screened *in vivo* for transduction efficiency or specificity. This approach has been leveraged to generate novel AAV vectors that can more effectively cross biological barriers and target specific cell types. An example is the identification of the AAV.7m8 variant which, following intravitreal injection, is capable of efficient gene delivery to all retina layers in both mice and primates (75). Similarly, SH10, an AAV6 variant, has increased tropism for glial cells following intravitreal delivery and has been shown to rescue retinal function in a rat model of RP (76,77).

Another strategy to achieve cell specificity is the use of cell type–specific promoters. Most gene therapy studies employ the use of strong ubiquitous promoters such as the cytomegalovirus or the chimeric chicken β-actin/cytomegalovirus enhancer/promoter (55). However, in some cases it may be desirable to limit expression to the target cell type to prevent unwanted side effects. For example, in the case of a gene editing approach, limiting expression of the Cas nuclease to the target cells reduces the risks associated with potential off-target editing events. Most gene therapies for IRDs are designed to target defects in either photoreceptors or the RPE. The rhodopsin kinase 1 and interphotoreceptor retinoid binding protein promoters can drive expression of transgenes in both cone and rod photoreceptors (78,79). For rod-specific expression, the rhodopsin promoter has been employed (64,80), while cone photoreceptor–specific expression can be achieved using cone arrestin, blue opsin, or red/green opsin promoters (68,81–83). Similarly, RPE-specific expression has been demonstrated using the *RPE65* or *VMD2* promoter (84,85), and specific expression can be achieved in ON-bipolar cells and Müller cells using cell type–specific promoters (86–88).

## Development of Ocular Gene Therapy Products

### Manufacturing Aspects: Production and Purification of Adeno Associated Viruses

Large-scale AAV manufacturing protocols for clinical use must consistently provide product that is safe and efficacious yet at a cost per dose that is acceptable to healthcare providers. The manufacturing requirement is for robust removal of product- and process-related impurities to meet agreed product specifications while retaining high recovery and titer of infectious AAV viruses.

Production of AAV vectors has been reviewed in detail by Ayuso *et al*. (89) and is briefly summarized here. Transient transfection protocols have been used for production of clinical-grade AAV in HEK293 human embryonic kidney cells and involve the co-transfection of three plasmids, one containing the expression cassette for the transgene, a second encoding the AAV regulatory and structural/capsid proteins, and a third that provides the viral helper functions essential for AAV replication. Alternatively, a packaging cell line can be constructed in which the genes encoding the AAV regulatory and structural/capsid proteins are integrated into the cell genome. To initiate AAV production, co-infection with a helper virus and a virus carrying the vector genome is required. Finally, a producer cell line can be used in which the transgene and the genes encoding the AAV regulatory and structural/capsid proteins are integrated into the cell genome. With producer cells, infection with only a helper virus such as Ad5 is required to initiate AAV production. Each of these approaches results in different challenges for purification.

With the transient transfection approach, each of the three different plasmids must be removed from the product, whereas use of a producer or packaging cell line introduces a helper and/or a vector-carrying virus that must be separated from the AAV product. The purification strategy will thus differ with the virus production approach that is adopted. The following discussion of process and product impurity issues assumes use of the transient expression process.

Process-related impurities arise from the manufacturing process. As with many cell-derived biotherapeutics the principal process-related impurities that contaminate the product stream are host cell proteins (HCPs), proteins from any serum- or other animal-derived products in the cell culture media, nucleic acids from host cells, and, in the case of AAV, non-transfected plasmid (90). HCPs and host cell nucleic acids are particularly an issue for AAV production since lysis of the producer cells is required to release AAV. These impurities, though, can generally be reduced to an acceptably low level during downstream processing. Another product-related concern arises from the possibility that the cell line may harbor adventitious agents that accumulate with the AAV product (91). Mitigation of this risk depends largely upon cell line characterization and validation during cell line development.

Product-related impurities arise from the physicochemical characteristics of the AAV product. They include empty AAV capsids that lack the viral genome and hence provide no therapeutic benefit while increasing the load of potentially immunogenic viral protein in the formulated product (92). AAV encapsidation of host cell nucleotides may also occur (90). These impurities all derive from the cell culture stage of manufacturing.

Aggregates of AAV viruses, an additional product-related impurity, are also undesirable as they reduce viral titer and hence product efficacy while also increasing the load of potentially immunogenic viral protein (90). Aggregates may be formed at multiple points in the manufacturing and purification processes and during subsequent formulation and storage. It is also conceivable that damage or oxidation of capsid proteins by inappropriate downstream processing conditions, at any stage in the process, might also impair the virus infection efficiency.

A general strategy for downstream purification of AAV that aims to mitigate these various impurity risks includes:

- Centrifugation and cell lysis: Cells are concentrated into a slurry by centrifugation and then lysed to release viruses by mechanical stress, hypertonic shock, or freeze-thaw procedures. Processing conditions targeted at the optimal release of viruses inevitably result in substantial contamination by HCPs and nucleic acids and may also cause damage to virions. Controls would thus include assays of infectious and viral particle titer, HCPs, and host cell and total DNA.
- Nucleic acid removal: To ensure a low level of nucleic acid contaminants, the lysate is digested with an endonuclease, commonly Benzonase® (Millipore Sigma; Burlington, MA). Additionally, evidence suggests that nucleic acids can mediate virus aggregation (93), presumably by bridging between cationic virions, and this effect is reduced by nuclease digestion. Nucleic acid material from host cells or plasmids that is encapsidated within AAV particles will be resistant to enzymatic digestion. Again, controls would include assays of infectious and viral particle titer (from which the ratio of full to empty virions can be calculated), HCPs, and host cell and total DNA.
- Solids removal: Centrifugation or microfiltration to remove cell fragments and debris prior to chromatographic purification.
- Affinity chromatography: Removal of HCPs and any serum protein impurities is efficiently achieved by affinity chromatography. Clinical preparations of AAV2 have been produced with heparin affinity chromatography using elution at elevated ionic strength (94,95). A wide range of AAV serotypes have been shown to be capable of affinity purification using AVB-Sepharose High Performance (GE Healthcare; Chicago, IL) (96), an adsorbent that is functionalized with single-domain antibody fragments that bind a commonly occurring epitope on the outer surface of the AAV capsid with high selectivity. Elution is conducted at low pH, and eluates must be neutralized promptly after collection as AAV is labile under highly acidic conditions. Infectious and viral particle titer, HCPs, and host cell and total DNA would be assayed, as well as the level of AAV aggregates.
- Ion-exchange chromatography: Formerly, the separation of genome-containing infectious AAV viruses from empty, non-infectious capsids was conducted by cesium chloride gradient ultracentrifugation. More recently, anion exchange chromatography has been shown to be able to separate full and empty capsids based on differences in electrical charge between the two particles due to the presence or absence of the viral genome (97). Elution is conducted with a high ionic strength buffer, which may also serve to reduce rates of virus aggregation. Infectious and viral particle titer, aggregates, HCPs, and host cell and total DNA would be assayed.
- Final polishing: Product stream polishing to further reduce levels of HCPs or other low molecular weight contaminants can be conducted using core-bead adsorbents. These adsorbents have a ligand-functionalized core to bind low molecular weight residual contaminants and exclude viruses with an outer shell that does not allow their passage.

Although this strategy reduces most process- and product-related impurities to clinically acceptable levels, it contains no specific step to remove AAV aggregates. Some level of aggregate clearance might arise from the affinity and ion-exchange chromatography stages due to differential rates of adsorption, but aggregates may reform during formulation and storage.

### Formulation Strategies

The majority of biological drug products (DPs), including gene therapy drugs, are administered by injection. For any injectable, a ready-to-use liquid formulation is usually the preferred dosage form because it is easier to manufacture compared with all other dosage forms and convenient from the end-user point of view. However, many biologics are not stable in an aqueous environment even under refrigeration and so are commonly stabilized by either drying (freeze-drying or occasionally spray- or vacuum-drying) or by freezing. In addition, various excipients (inactive ingredients) are commonly used to minimize degradation during freezing/storage/thawing and freeze-drying/storage/reconstitution. A typical biological DP contains several excipients, including a buffer, lyo/cryo protector, tonicity agent, and surfactant (98,99). Formulation development usually starts with identification of the pH associated with maximal stability and selection of a buffer with an adequate buffering capacity in the target pH range. Common advice for formulating proteins includes avoiding a pH close to the isoelectric point of a particular active ingredient and minimization of ionic strength. However, AAV formulations could require use of a higher ionic strength in order to prevent aggregation, as discussed below.

Potential pH changes during freezing should also be taken into consideration. There are three common mechanisms of freeze-induced pH changes, including crystallization of the buffer components, as commonly observed with sodium phosphate buffer; temperature dependence of pH; and change in the apparent acid dissociation constant (pKa) as a result of a decrease in the polarity of the amorphous (liquid) phase due to freeze-concentration. The low-temperature behavior of pharmaceutically relevant buffers, impact of other solutes on the pH of frozen systems, and recommendations for buffer selection are all reviewed in Wu *et al*. (100). Polyhydroxy compounds, such as carbohydrates and sugar alcohols, are usually used as lyo/cryo protectors, with trehalose, sucrose, and sorbitol representing the most common choices. Non-ionic surfactants, e.g. polysorbate or a poloxamer, are also commonly included in the formulation to minimize stresses due to exposure to different surfaces during manufacturing and to improve protein recovery at the point of use. This section is focused on AAV-based products, although general principles are applicable to other virus-based gene delivery systems.

Gene delivery products are typically stored as frozen solutions. DP design for AAV is similar to that of other biologics, with the majority of formulations containing buffer, tonicity agent, cryoprotector, and surfactant. Although the purity of a vector is mainly controlled during DS manufacture (90), the DP production and storage can have an impact on the potency and also introduce undesirable modified species of AAV, including aggregates, oxidized virus particles, and other degraded species. Impurities represent potential immunotoxicity risks, while aggregates could also impact biodistribution and cause inconsistency in the *in vivo* functional activity of the AAV, as discussed by Wright *et al*. (93). It was suggested, for example, that delivery of an AAV vector to hepatocytes would require vector particles to pass through the endothelial lining of hepatic sinusoids to reach target hepatocytes. Considering that the endothelial cell lining contains intercellular gaps of 0.1–1 μm, passage of aggregated AAV particles could be inhibited. The majority of the discussions in the literature on DP-related impurities are focused on virus aggregation. Some of the techniques used to study the aggregation of AAV are discussed below under Properties and Characterization of Vectors.

**Fig. 2:**
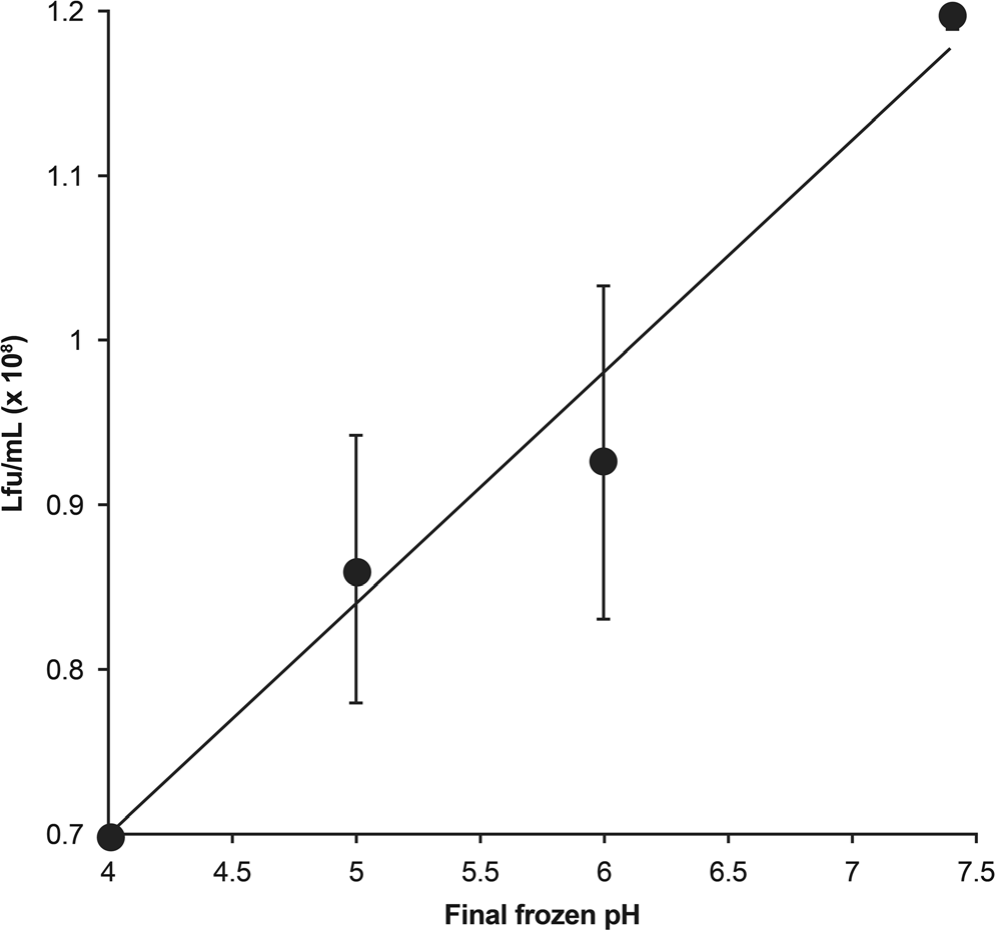
Adeno-associated virus (AAV) stability with respect to final frozen pH (correlation coefficient, r^2^ = 0.97). *Lfu represents lac-forming unit. Reprinted by permission from Springer Nature: Springer Publishing Company, Gene Therapy. Croyle MA, Cheng X, Wilson JM. Development of formulations that enhance physical stability of viral vectors for gene therapy*. *© 2001. 2001* (101).

Many biologics are sensitive to pH, and relationships between solution pH and stability (the pH stability profile) are usually established during preformulation/early formulation development in order to identify the pH range associated with maximal stability. Stability of AAV during freeze-thaw was reported to be dependent on the pH of the frozen solution (when freezing-induced pH changes are taken into consideration), with stability improved when the pH was increased from 4 to 7 (Fig. 2) (101). Structural changes in the AAV capsid related to pH were also reported. According to Venkatakrishnan *et al*. (102), the α-helical structure of VP1u (a unique 137–amino acid N-terminal region of VP1) in AAV1 and AAV6 was gradually lost when pH was decreased from 7.5 to 4.0; this structural change was reversed when pH was increased back to 7.5. The VP3 common region was unperturbed by pH changes. Furthermore, capsid integrity was maintained in the entire pH range of 7.5 to 4, based on negative-stain electron microscopy. We note that although the pH-induced partial unfolding of VP1u capsid protein is reversible, it is possible that the unfolded regions could be prone to hydrophobic aggregation. This would be consistent with the observed pH trend in the freeze-thaw stability of AAV (Fig. 2). Note however that an opposite pH trend was reported for solubility of AAV2 (concentration approximately 0.1 mg/mL), with solubility monotonically increasing with the decrease in pH from 10 to 4.5 (103).

**Fig. 3:**
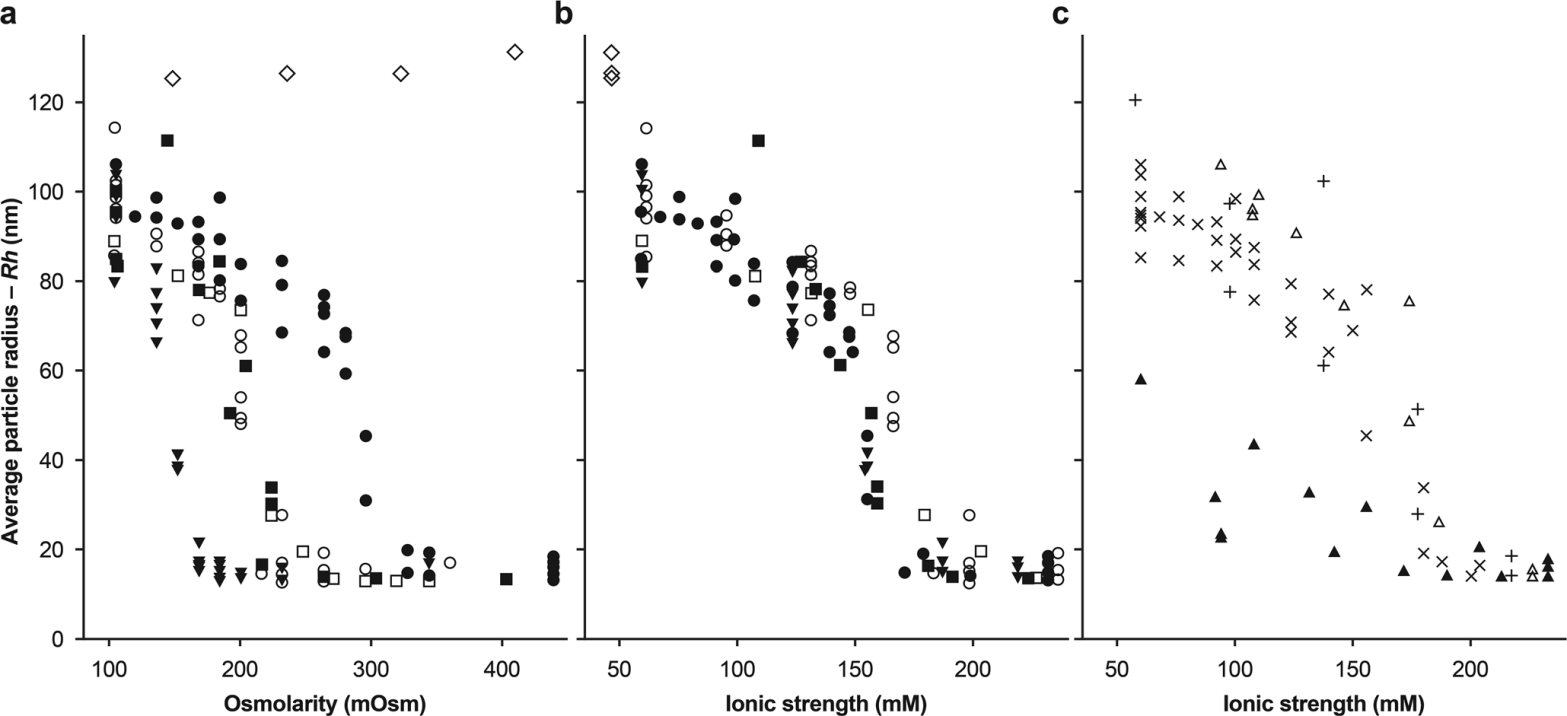
Dependence of adeno-associated virus serotype 2 (AAV2) vector aggregation on osmolarity (**a**), ionic strength (**b**), and purification method (**c**). The average particle radius of the AAV2-FIX vector was measured by dynamic light scattering following vector dilution in varying concentrations of excipients buffered with 10 mM sodium phosphate, pH 7.5. (**a**, **b**) Filled circles: sodium chloride; open circles: sodium citrate; filled squares: sodium phosphate; open squares: sodium sulfate; inverted filled squares: magnesium sulfate; open diamonds: glycerol. Vector was purified by method 3 (chromatography plus CsCl gradient). (**c**) The vector was purified by one of three different methods as the ionic strength was adjusted with NaCl. + symbols: method 1 (double CsCl gradient); open triangles: method 2 (cation exchange chromatography); filled triangles: method 2 plus nuclease digestion; x symbols: method 3. *Republished from Wright et al*. (93) *under the terms of the CC BY-NC-ND 4.0 license (**http://creativecommons.org/licenses/by-nc-nd/4.0/**).*

The impact of ionic strength on AAV aggregation was studied by Wright *et al*. (93) using the “dilution-stress” method. In that study, highly concentrated AAV samples were diluted with different excipients, and aggregation of the diluted samples was measured by dynamic light scattering. Charged excipients (inorganic salts and amino acids) were found to prevent aggregation. The inhibition of AAV aggregation by ionic excipients depends on their type and concentration, with multi-charged salts being effective at concentrations corresponding to 180–220 mOsm *versus* 300–320 mOsm for NaCl and amino acids. It was further shown that the inhibition of AAV aggregation by salts was related to the ionic strength of the solution rather than the osmolarity (Fig. 3). Figure 3a and b show particle sizes (by dynamic light scattering) for the same formulations (same Y axis) but plotted as a function of osmolarity in Fig. 3a and as a function of ionic strength in Fig. 3b and c. There are no correlations between osmolarity and virus aggregation, whereas the relationship between ionic strength and particle size (which reflects aggregation) is obvious. An important conclusion from this study was that multivalent salts require lower concentrations and osmolarity than monovalent salts such as NaCl to prevent aggregation of AAV (e.g.*,* magnesium sulfate at ~200 mOsm was as effective as 350 mOsm of NaCl in stabilization of AAV against aggregation). Improvement in solubility by Mg^2+^ (20 mM) was also reported (pH 4.5 to 7.5) (103); however, no improvement in solubility with a multi-charge anion (citrate^3−^) was observed.

The AAV purification method also was observed to have a significant effect on aggregate formation, as illustrated in Fig. 3c (93). Removal of DNA impurities by treatment with nuclease resulted in reduction in aggregation even at lower ionic strength. These results point to electrostatic attraction as the main driving force for the aggregation observed, in which DNA is sorbed on the virus capsid particle. DNA and the capsid proteins have different acid dissociation constants, and the difference in charges creates electrostatic attraction and ionic bridges between the sorbed DNA and the DNA-free part of another virus particle.

Non-ionic surfactants (Pluronic® F68 [(BASF, Mount Olive, NJ, USA] and polysorbate PS80) and polyhydroxy compounds were ineffective in the prevention of the aggregation, at least at the concentrations studied (93). Maximal concentrations tested were 10% w/v, 1% w/v, and 5% w/v for F68, PS80, and polyhydroxy compounds (glycerol, mannitol, sorbitol, sucrose, trehalose), respectively. In other reports, however, non-ionic excipients were found to be somewhat more effective in stabilization of AAV against aggregation. For example, prevention of AAV aggregation by 25% glycerol was reported (103), although such a high concentration is not practical in a DP. In addition, a lower concentration of glycerol (3%) was found to be sufficient to eliminate AAV aggregation on a solid surface (104). Also, it was reported that a non-ionic surfactant (beta-octyl glucopyranoside at 0.01–0.5%, below the critical micelle concentration) reduced aggregation (103).

AAV DP manufacture employs standard steps that are used for all parenteral products. The typical manufacturing process for an injectable product includes sterilizing filtration with a 0.22-μm filter, aseptic filling into a sterile container (usually glass or plastic sterile vials), sealing with a rubber stopper, and crimping with an aluminum shell. As with any product with a low concentration of an active ingredient (a typical concentration range for AAV is approximately 0.01–0.1 mg/mL, corresponding to 1E12 to 1E13 vg/mL), there is a risk of losing the active ingredient due to its sorption on different surfaces. Indeed, significant (30–40%) losses of AAV were reported during filtration, although the losses were minimized with formulation optimization (93). Furthermore, Xie *et al*. (103) reported major (up to 80%) losses of AAV during centrifugal concentration because of sorption of the virus on the membrane. Use of polyethylene glycol (12–20 kDa) at 5–20% reduced losses to 30%, whereas a high concentration of glycerol (up to 25%) further minimized sorption.

**Fig. 4:**
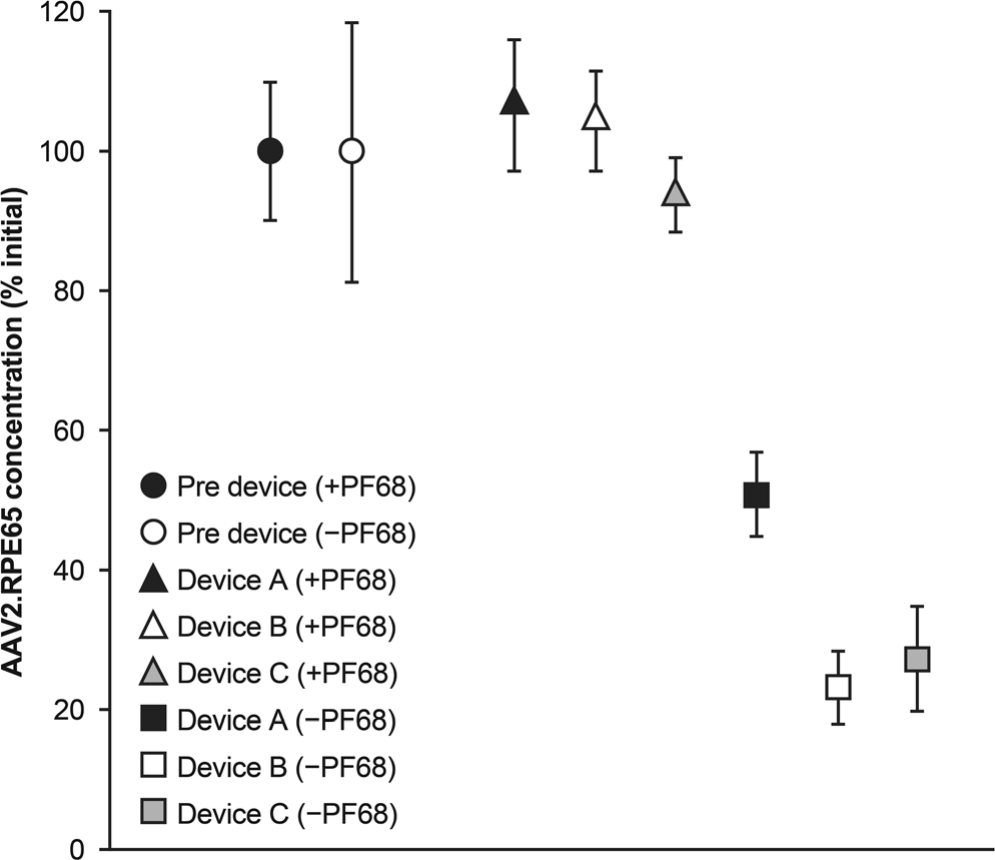
Recovery of adeno-associated virus serotype 2 (AAV2) following dilution and passage through the administration device. Stock AAV2-RPE65 vector diluted to 1×10^11^ vg/mL in phosphate-buffered saline with (+PF68) or with (−PF68). Pluronic 68 0.001% was drawn into 1-mL syringes, and the vector was passed through device A, B, or C. *Republished from Bennicelli et al*. (105) *under the terms of the CC BY-NC-ND 4.0 license (**http://creativecommons.org/licenses/by-nc-nd/4.0/**).*

Significant losses of virus were also observed under simulated in-use conditions. As shown in Fig. 4, up to 80% of AAV was lost after passing through different delivery devices when formulations without a surfactant were used (105). Remarkably, a formulation with the surfactant Pluronic F68 (0.001%) showed essentially 100% preservation of the virus. In addition, Sommer *et al*. (106) reported that the addition of polysorbate 80 or Pluronic F68 at concentrations of 0.01 and 0.001%, respectively, could prevent losses of the vector.

In a typical DP manufacturing process, an AAV formulation can be exposed to freeze-thaw (e.g., when the DS is stored in the frozen state and is thawed in preparation of the DP production), and short-term storage is in the liquid state. Freeze-thaw and storage at 5°C was shown to cause AAV aggregation, although the effect was formulation-dependent (93). In the best formulation studied, no aggregation was observed after 5 days at 5°C and after one freeze-thaw cycle (−80°C), while aggregation was observed after five freeze-thaw cycles (93). It should be noted that AAV freeze-thaw stability can be impacted by freeze-induced pH changes; the pH changes depend on the type and concentration of buffer and the presence of other excipients (100).

**Fig. 5:**
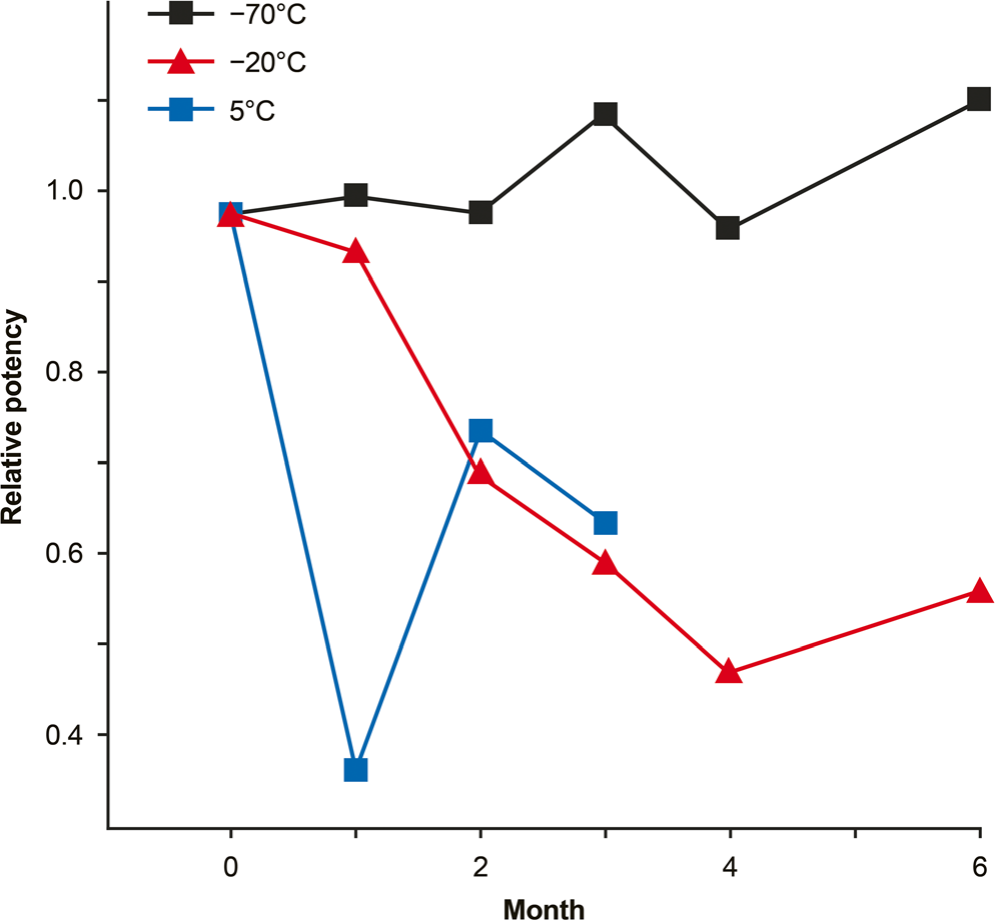
Potency of liquid formulation for an investigational protein drug product during storage at different temperatures (data from EY Shalaev, “Role of Ice in Destabilization of Proteins”, presented at the AAPS National Biotechnology Conference, Boston, MA, May 18, 2016).

Finished AAV DP is usually stored as a frozen solution. Although a more convenient temperature range for frozen storage is −15 to −25°C, keeping water-based biologics under these conditions may represent a significant stability risk. Aqueous biopharmaceuticals (including AAV formulations) are not completely frozen at −15 to −25°C. They usually consist of at least two phases, ice and freeze-concentrated solution (107). The freeze-concentrated solution, which contains all the solutes, an active ingredient (e.g., AAV2), and unfrozen water, is squeezed between ice crystals. This freeze-concentrated solution is indeed liquid at −20°C and solidifies (forming a glassy state) below its corresponding glass transition temperature. In a typical biological system, the glass transition temperature of the freeze-concentrate is −35 to −50°C, or even lower. There are multiple destabilization mechanisms associated with −20°C storage, such as greatly increased concentrations of all ingredients, which could lead to acceleration of bimolecular (e.g., aggregation) processes, pH changes, an increase in oxygen concentration, an extensive ice/solution interface, and crystallization of a cryoprotector. Many cases of chemical and physical instability in partially frozen systems have been reported in the literature (108–116). An additional example of major destabilization of an investigational protein drug is provided in Fig. 5, showing ~40% loss of potency after 3 months at −20°C. Overall, storage at lower temperatures, e.g., below −65°C, should be used for aqueous gene therapy products as a default.

While AAV DP can be quite stable in the frozen state, shipping and storage of frozen biologics could present some practical challenges. Therefore, AAV formulations that are stable above 0°C are desirable. There have been some efforts to evaluate freeze-drying as well as long-term storage of liquid AAV formulations. The impact of freeze-drying on AAV was studied by Croyle *et al*. (101). Some loss of titer (0.3 log) was observed after lyophilization of AAV formulations with phosphate potassium buffer, as well as with a formulation containing 0.4% sucrose, 0.4% mannitol, and protamine. The latter formulation was stable after storage for 3 months at 25°C. Interestingly, a liquid formulation of the same virus with a similar composition (0.4% sucrose, 0.4% mannitol, 0.001% sorbitan monolaurate [Span 20], and 0.1% protamine) was also quite stable, with only 0.1 log titer loss after 6 months at both 4° and 25°C. Similar long-term liquid stability was also reported by Wright *et al*. (117), where AAV vector in neutral phosphate buffered saline with 5% sorbitol and 0.1% polysorbate 80 showed no significant loss of transduction activity after 1 year at 2–8°C. On the other hand, up to 40% loss in transgene expression after 7 weeks at 4°C was observed for AAV1 virus diluted in phosphate-buffered saline containing 0.5 mM of MgCl_2_ (118).

### Properties and Characterization of Vectors

Gene therapy products represent a novel and complex class of pharmaceutical products that require sophisticated technical and scientific assessment during the drug development. To ensure the products’ safety, integrity, potency, and purity, the application of key advanced characterization technologies is essential to assess the quality of all components (i.e., vectors, reagents, excipients) used for manufacturing of the final product formulation. The Center for Biologics Evaluation and Research at the FDA has implemented a stepwise approach to product characterization in compliance with current Good Manufacturing Practice (cGMP), which is described in the FDA guidelines for human gene therapy (119). All materials and components used for manufacture of a gene therapy product, e.g., vectors, reagents, and excipients, should be adequately characterized (119).

Preformulation and formulation characterization is performed in the early stage of product development. A full product characterization helps to determine the impact of the manufacturing process, process parameters, and excipients on the quality of the gene therapy products.

Vector concentration is one of the quality attributes of gene therapy products. A standard method for measurement of the AAV concentration in both DS and DP is quantitative polymerase chain reaction. D’Costa *et al*. (120) and Neuberger *et al*. (121) described the use of quantitative polymerase chain reaction and its advantages for quantification of AAV. Another critical quality attribute for AAV-based gene therapy products is the empty/full capsids ratio, which is traditionally measured using density-based analytical centrifugation. Sommer *et al*. (122) further showed that ultraviolet absorbance of denatured AAV vectors can be utilized to quantify vector genomes and the content of capsid proteins in solutions.

A variety of advanced analytical technologies such as small angle X-ray scattering, transmission electron microscopy (TEM), cryo-TEM, synchrotron X-ray diffraction, differential scanning calorimetry, ion exchange chromatography, differential scanning fluorimetry (DSF), circular dichroism (CD) spectrometry, and others have been used in the literature to characterize vectors used for gene therapy products. For example, Xie *et al*. (103) characterized AAV2 by TEM after large-scale purification and showed a homogenous preparation of full AAV2 particles on the nanometer scale. Additionally, semiconductor quantum dots (QDs) have been used for long-term live-cell imaging and detection of viral particles with high sensitivity. Joo *et al*. (123) described labeling AAV2 with QDs to visualize and monitor viruses within living cells. The viral infectivity of AAV2 was maintained by the mild conditions used for the QD conjugation reaction (123).

**Fig. 6:**
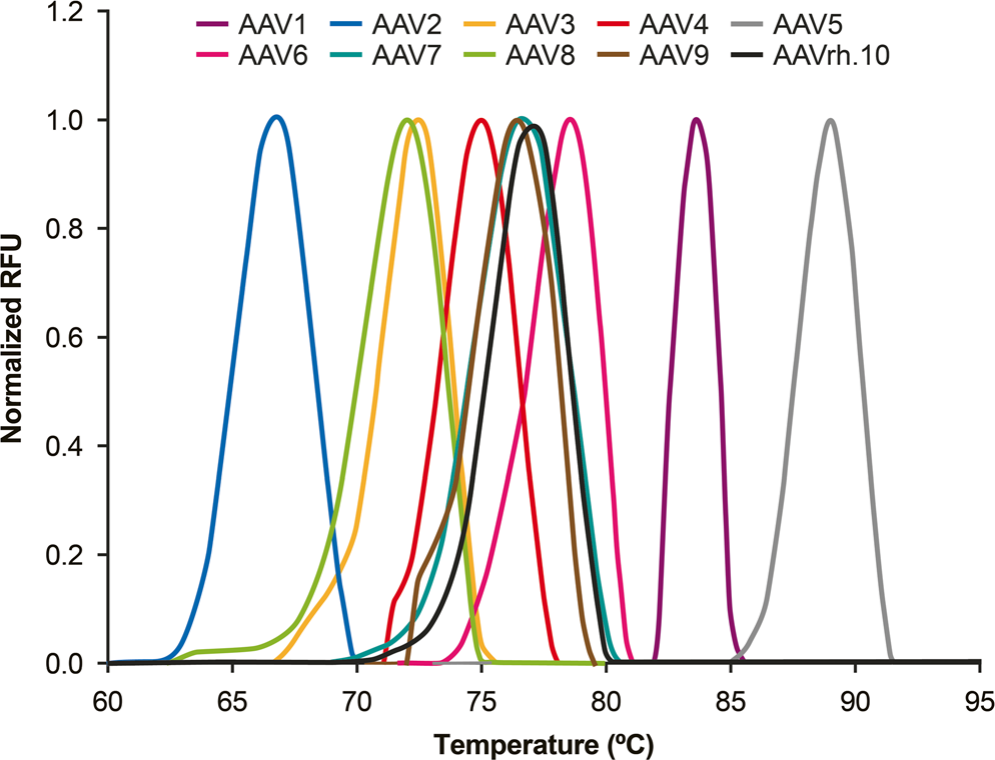
Thermal profiles of rAAV serotypes 1 to 9 and rAAVrh.10 produced by DSF analysis. The DSF spectra display normalized RFUs (relative fluorescence units) *vs.* temperature. A representative DSF spectrum is shown for each rAAV serotype. *Republished from Bennett et al*. (124) *under the terms of the CC BY-NC-ND 4.0 license (**http://creativecommons.org/licenses/by-nc-nd/4.0/**).*

Bennett *et al*. (124) suggested the application of DSF to determine melting temperatures for AAV serotype identification (Fig. 6). DSF is a rapid, robust, and cost-effective analytical method that requires small quantities of purified AAV capsids (~10^11^ particles in ~25 μL). The DSF method evaluates the thermal profile of vectors, where the normalized relative fluorescence units (RFU) are plotted *versus* temperature, and the melting point is determined as the temperature that corresponds to the apex at a maximum RFU (Fig. 6). The study by Bennett *et al*. (124) on AAV1–9 and AAVrh.10 showed that those AAV serotypes exhibited distinct, specific melting temperatures in buffered formulations commonly used in clinical trials.

**Fig. 7:**
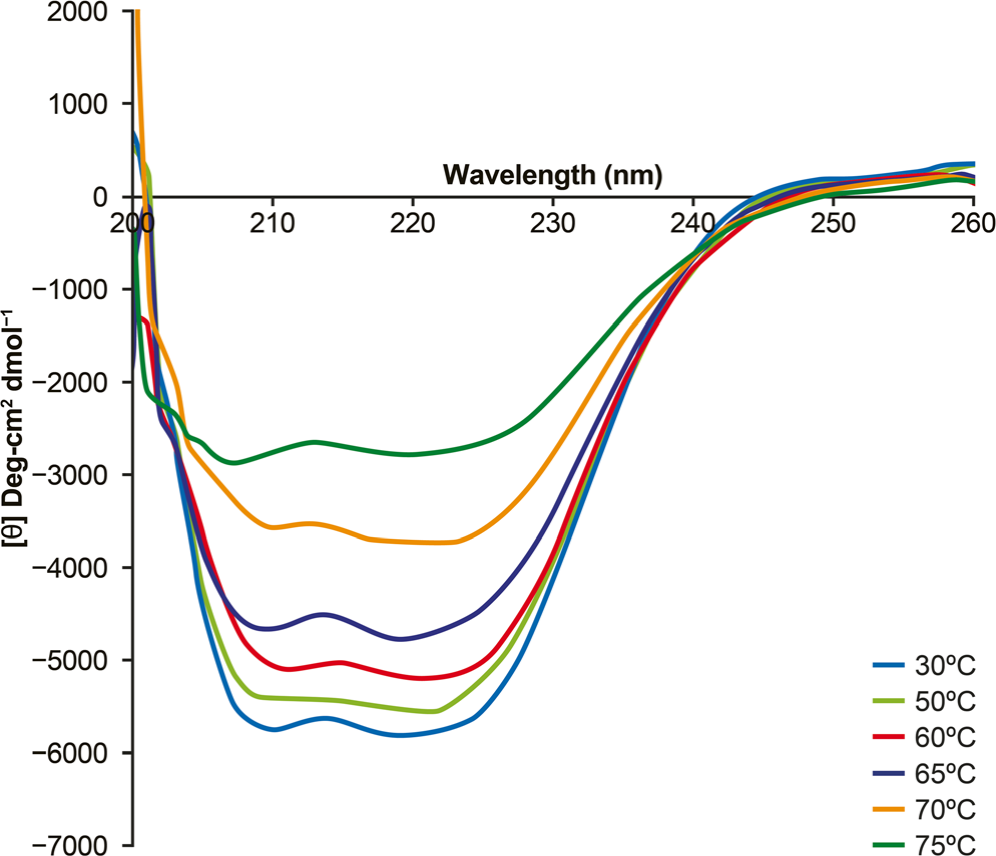
Example of circular dichroism (CD) spectra of AAV serotype 1 (AAV1) viral-linked proteins at different temperatures. A clear α-helical propensity can be seen for AAV1. This helical signal is lost as temperature increases. *Reproduced from Venkatakrishnan et al*. *J Virol. 2013;87:4974–84. doi* 10.1128/JVI.02524-12 (102) *with permission from American Society for Microbiology. © 2013.*

Venkatakrishnan *et al*. (102) used biophysical and computational approaches to study the structure of the viral protein VP1 in AAV serotypes 1–12. CD spectrometry and cryo-TEM were used to examine the effects of temperature and the pH values encountered during endocytic transport on the conformation of the AAV capsids. CD spectrometry indicated that in solution the viral protein VP1u in AAV1 and AAV6 exhibits a well-ordered α-helical secondary structure. Gradual loss of this α-helical structure was seen when the pH decreased from 7.5 to 4.0, and this loss was reversed when the pH went back up to 7.5. CD spectroscopy experiments confirmed the effect of temperature on the ordered α-helical structure of the viral-linked proteins of AAV1 (Fig. 7) (102). On the other hand, negative-stain electron microscopy visualization suggested that AAV1 capsids maintain their integrity upon the pH treatment, consistent with earlier observations of the maintenance of structural integrity of AAV8 capsids in spite of pH changes in the endocytic pathway (102).

As discussed above, one aspect of the physical instability of vectors is aggregation. Wright *et al*. (93) studied aggregation of rAAV2 and the effect of aggregation on vector performance. Dynamic light scattering can be employed to determine the aggregation of AAV with high sensitivity. It requires very small sample volumes (20 μL) and provides a semi-quantitative measure of AAV aggregation. The size of aggregates or nano-aggregated AAVs can also be measured by applying small-angle X-ray scattering. A potential advantage of this technique is that it can be applied to samples that are not optically transparent, such as frozen solutions and freeze-dried powders.

The development of novel and sophisticated state-of-the-art technologies with higher resolution and applications of these new technologies for characterization of biologics, including vectors, will offer a better scientific understanding of the physicochemical properties, purity, and stability of the vector-based formulations used for human gene therapy products in the future.

### Regulatory Perspective

In the United States, the Center for Biologics Evaluation and Research of the FDA regulates human gene therapy products. The rigorous preclinical and clinical assessments that are required for gene therapy products are outlined in regulatory guidance documents. Since the first FDA guideline on human gene therapy was published in April 2008 (119), significant progress has been made in the field of gene therapy. In July 2018, the FDA unveiled update draft guidance documents on human gene therapy that outline the current regulatory perspective. The six new FDA guidelines focus on the following areas:

I. Chemistry, Manufacturing and Control (CMC) Information for Human Gene Therapy Investigational New Drug Applications (INDs) (125)
II. Human Gene Therapy for Retinal Disorders (126)
III. Testing of Retroviral Vector-Based Human Gene Therapy Products for Replication Competent Retrovirus During Product Manufacture and Patient Follow-up (127)
IV. Human Gene Therapy for Hemophilia (128)
V. Human Gene Therapy for Rare Diseases (129)
VI. Long Term Follow-Up After Administration of Human Gene Therapy Products (130)

The first three documents above contain the most relevant guidance applicable to ocular gene therapy. The draft guidance on CMC information for human gene therapy INDs (125) is intended to update the April 2008 guidance (119). Its aim is to “inform sponsors of gene therapy products with regulatory expectations on CMC information” and data that are required “to assure the product safety, integrity, quality, purity, and strength (including potency) of an investigational gene therapy product” (125). The new guideline provides detailed recommendations on the description of the DS and DP, including the physical, chemical, or biological characteristics, manufacturing process and controls, and testing information, for the purpose of ensuring the DS and DP meet acceptable limits for identity, strength (potency), quality, and purity. For example, information needs to be provided to illustrate the structure and structural elements of gene therapy products utilizing either viral vectors or bacterial vectors. Another requirement is a complete description of all procedures used for gene modification when the product consists of genetically modified cells (125). It should be noted that these six new regulatory guidelines issued by FDA are all in ‘draft’ format and may be subject to change in the future. The European Medicines Agency has also recently (March 2018) updated its guidance on the quality, nonclinical, and clinical aspects of gene therapy medicinal products (131). A comparative review of the content of the European Medicines Agency guideline *versus* the FDA guidance on the CMC information for human gene therapy is not within the scope and objectives of this review article. Nor do we review the status of the regulatory guidelines for human gene therapy in countries outside the United States or European Union.

One of the concerns for gene therapy products is the potential for virus or vector shedding, especially when the used virus or gene therapy vectors are considered capable of replication or persisting in patients over extended periods of time, as suggested by limited findings in patient excreta. Consequently, human-to-human transmission as a potential outcome of shedding may become an important public health issue. The FDA guideline entitled: “Design and Analysis of Shedding Studies for Virus or Bacteria-Based Gene Therapy and Oncolytic Products” provides guidance on vector shedding studies, including both how and when shedding data should be collected and how shedding data can be used to assess the potential for transmission to untreated individuals (132). The European Medicines Agency has a similar guideline on vector shedding entitled: “ICH Considerations - General Principles to Address Virus and Vector Shedding” (133). Currently there is no harmonized guideline on vector shedding. The International Council for Harmonisation of Technical Requirements for Pharmaceuticals for Human Use (ICH) endorsed a guideline topic, “Virus and Gene Therapy Vector Shedding and Transmission” in September 2009, which was assigned as M6. Later, in April 2011, development of the M6 guideline was rejected following discussion by the ICH Steering Committee that concluded: “due to the state of the science and related resource allocation the vector shedding would not be supported as a topic for harmonization” (134). Vector shedding was investigated in patients’ tears, serum, and whole blood as part of the clinical assessment for the first ocular gene therapy product, voretigene neparvovec-ryzl, during the phase III clinical trial (135).

## Conclusions and Outlook

The approval in 2017 of voretigene neparvovec-rzyl marked a key milestone in the evolution of gene therapy as a modality for treatment of ocular diseases. We anticipate that investment in gene-based therapies will continue for both IRDs and other ocular diseases, and indeed, the clinical and preclinical pipeline is robust with dozens of programs in development. Although AAV is now firmly established as the leading vector technology, lentivirus also remains in use and can be considered for delivery of larger transgenes. Efforts in non-viral delivery also continue, with the EyeCET electroporation technology currently being evaluated in a phase I/II study. Continued optimization of AAV by both directed evolution and rational design will likely lead to additional new vectors with improved attributes, including more efficient transduction of target tissues following delivery using less invasive routes of administration (e.g., intravitreal or suprachoroidal injection). The production, formulation, and characterization of AAV-based products generally leverages know-how and methods already established for biologics. The general regulatory expectations in the United States and European Union seem to be similar. Nonetheless, we anticipate that formulation and analytical tools will also continue to evolve as use of AAV becomes more prevalent, and that regulatory expectations will also continue to be clarified as additional products progress through development and approval. With the recent scientific advances and clinical successes, gene therapy has emerged as a powerful approach with the potential to offer long-lasting therapeutic benefits to address unmet medical needs. We expect continued advances in translating gene therapy into an important modality for the treatment of ocular disorders.

## Abbreviations

AAV: Adeno-associated virus
AMD: Age-related macular degeneration
CD: Circular dichroism
CMC: Chemistry, manufacturing, and controls
DP: Drug product
DS: Drug substance
DSC: Differential scanning calorimetry
DSF: Differential scanning fluorimetry
FDA: United States Food and Drug Administration
GMP: Good manufacturing practice
HCP: Host cell protein
ICH: International Council for Harmonisation of Technical Requirements for Pharmaceuticals for Human Use
IND: Investigational new drug application
IRD: Inherited retinal disease
kb: Kilobase
LCA: Leber congenital amaurosis
LHON: Leber hereditary optic neuropathy
ORF: Open reading frame
PLA: Poly(lactic acid)
PLGA: Poly(lactic-*co*-glycolic acid)
QD: Quantum dot
rAAV: Recombinant AAV
RP: Retinitis pigmentosa
RPE: Retinal pigment epithelium
RPE65: Retinal pigment epithelium–specific 65-kDa protein
sFlt-1: Fms-like tyrosine kinase-1
SNP: Single-nucleotide polymorphism
TEM: Transmission electron microscopy
VEGF: Vascular endothelial growth factor
XLRP: X-linked retinitis pigmentosa
XLRS: X-linked retinoschisis

## References

[1] Dunbar CE, High KA, Joung JK, Kohn DB, Ozawa K, Sadelain M (2018). Gene therapy comes of age. Science.

[2] Bainbridge JW, Smith AJ, Barker SS, Robbie S, Henderson R, Balaggan K, Viswanathan A, Holder GE, Stockman A, Tyler N, Petersen-Jones S, Bhattacharya SS, Thrasher AJ, Fitzke FW, Carter BJ, Rubin GS, Moore AT, Ali RR (2008). Effect of gene therapy on visual function in Leber's congenital amaurosis. N Engl J Med.

[3] Hauswirth WW, Aleman TS, Kaushal S, Cideciyan AV, Schwartz SB, Wang L, Conlon TJ, Boye SL, Flotte TR, Byrne BJ, Jacobson SG (2008). Treatment of leber congenital amaurosis due to *RPE65* mutations by ocular subretinal injection of adeno-associated virus gene vector: short-term results of a phase I trial. Hum Gene Ther.

[4] Maguire AM, Simonelli F, Pierce EA, Pugh EN, Mingozzi F, Bennicelli J, Banfi S, Marshall KA, Testa F, Surace EM, Rossi S, Lyubarsky A, Arruda VR, Konkle B, Stone E, Sun J, Jacobs J, Dell'Osso L, Hertle R, Ma JX, Redmond TM, Zhu X, Hauck B, Zelenaia O, Shindler KS, Maguire MG, Wright JF, Volpe NJ, McDonnell JW, Auricchio A, High KA, Bennett J (2008). Safety and efficacy of gene transfer for Leber's congenital amaurosis. N Engl J Med.

[5] Naldini L (2009). Medicine. A comeback for gene therapy. Science.

[6] Boye SE, Boye SL, Lewin AS, Hauswirth WW (2013). A comprehensive review of retinal gene therapy. Mol Ther.

[7] Cideciyan AV, Hauswirth WW, Aleman TS, Kaushal S, Schwartz SB, Boye SL, Windsor EA, Conlon TJ, Sumaroka A, Pang JJ, Roman AJ, Byrne BJ, Jacobson SG (2009). Human *RPE65* gene therapy for Leber congenital amaurosis: persistence of early visual improvements and safety at 1 year. Hum Gene Ther.

[8] Jacobson SG, Cideciyan AV, Ratnakaram R, Heon E, Schwartz SB, Roman AJ, Peden MC, Aleman TS, Boye SL, Sumaroka A, Conlon TJ, Calcedo R, Pang JJ, Erger KE, Olivares MB, Mullins CL, Swider M, Kaushal S, Feuer WJ, Iannaccone A, Fishman GA, Stone EM, Byrne BJ, Hauswirth WW (2012). Gene therapy for leber congenital amaurosis caused by *RPE65* mutations: safety and efficacy in 15 children and adults followed up to 3 years. Arch Ophthalmol.

[9] Simonelli F, Maguire AM, Testa F, Pierce EA, Mingozzi F, Bennicelli JL, Rossi S, Marshall K, Banfi S, Surace EM, Sun J, Redmond TM, Zhu X, Shindler KS, Ying GS, Ziviello C, Acerra C, Wright JF, McDonnell JW, High KA, Bennett J, Auricchio A (2010). Gene therapy for Leber's congenital amaurosis is safe and effective through 1.5 years after vector administration. Mol Ther.

[10] Testa F, Maguire AM, Rossi S, Pierce EA, Melillo P, Marshall K, Banfi S, Surace EM, Sun J, Acerra C, Wright JF, Wellman J, High KA, Auricchio A, Bennett J, Simonelli F (2013). Three-year follow-up after unilateral subretinal delivery of adeno-associated virus in patients with Leber congenital Amaurosis type 2. Ophthalmology.

[11] Bennett J, Ashtari M, Wellman J, Marshall KA, Cyckowski LL, Chung DC, McCague S, Pierce EA, Chen Y, Bennicelli JL, Zhu X, Ying GS, Sun J, Wright JF, Auricchio A, Simonelli F, Shindler KS, Mingozzi F, High KA (2012). Maguire AM. AAV2 gene therapy readministration in three adults with congenital blindness. Sci Transl Med.

[12] Bainbridge JW, Mehat MS, Sundaram V, Robbie SJ, Barker SE, Ripamonti C, Georgiadis A, Mowat FM, Beattie SG, Gardner PJ, Feathers KL, Luong VA, Yzer S, Balaggan K, Viswanathan A, de Ravel TJ, Casteels I, Holder GE, Tyler N, Fitzke FW, Weleber RG, Nardini M, Moore AT, Thompson DA, Petersen-Jones SM, Michaelides M, van den Born LI, Stockman A, Smith AJ, Rubin G, Ali RR (2015). Long-term effect of gene therapy on Leber's congenital amaurosis. N Engl J Med.

[13] Jacobson SG, Cideciyan AV, Roman AJ, Sumaroka A, Schwartz SB, Heon E, Hauswirth WW (2015). Improvement and decline in vision with gene therapy in childhood blindness. N Engl J Med.

[14] Russell S, Bennett J, Wellman JA, Chung DC, Yu ZF, Tillman A, Wittes J, Pappas J, Elci O, McCague S, Cross D, Marshall KA, Walshire J, Kehoe TL, Reichert H, Davis M, Raffini L, George LA, Hudson FP, Dingfield L, Zhu X, Haller JA, Sohn EH, Mahajan VB, Pfeifer W, Weckmann M, Johnson C, Gewaily D, Drack A, Stone E, Wachtel K, Simonelli F, Leroy BP, Wright JF, High KA, Maguire AM (2017). Efficacy and safety of voretigene neparvovec (AAV2-hRPE65v2) in patients with *RPE65*-mediated inherited retinal dystrophy: a randomised, controlled, open-label, phase 3 trial. Lancet.

[15] MacLaren RE, Groppe M, Barnard AR, Cottriall CL, Tolmachova T, Seymour L, Clark KR, During MJ, Cremers FP, Black GC, Lotery AJ, Downes SM, Webster AR, Seabra MC (2014). Retinal gene therapy in patients with choroideremia: initial findings from a phase 1/2 clinical trial. Lancet.

[16] Solinís MÁ, del Pozo-Rodríguez A, Apaolaza PS, Rodríguez-Gascón A (2015). Treatment of ocular disorders by gene therapy. Eur J Pharm Biopharm.

[17] Farrar GJ, Carrigan M, Dockery A, Millington-Ward S, Palfi A, Chadderton N, Humphries M, Kiang AS, Kenna PF, Humphries P (2017). Toward an elucidation of the molecular genetics of inherited retinal degenerations. Hum Mol Genet.

[18] Black JR, Clark SJ (2016). Age-related macular degeneration: genome-wide association studies to translation. Genet Med.

[19] Khan AO (2011). Genetics of primary glaucoma. Curr Opin Ophthalmol.

[20] Eghrari AO, Riazuddin SA, Gottsch JD (2015). Fuchs corneal dystrophy. Prog Mol Biol Transl Sci.

[21] Daiger SP, Sullivan LS, Bowne SJ (2013). Genes and mutations causing retinitis pigmentosa. Clin Genet.

[22] RetNet™ Retinal Information Network. Available from: https://sph.uth.edu/retnet/. Accessed 20 Nov 2018.

[23] Veleri S, Lazar CH, Chang B, Sieving PA, Banin E, Swaroop A (2015). Biology and therapy of inherited retinal degenerative disease: insights from mouse models. Dis Model Mech.

[24] Stone EM, Andorf JL, Whitmore SS, DeLuca AP, Giacalone JC, Streb LM, Braun TA, Mullins RF, Scheetz TE, Sheffield VC, Tucker BA (2017). Clinically focused molecular investigation of 1000 consecutive families with inherited retinal disease. Ophthalmology.

[25] Ferrari S, Di Iorio E, Barbaro V, Ponzin D, Sorrentino FS, Parmeggiani F (2011). Retinitis pigmentosa: genes and disease mechanisms. Curr Genomics.

[26] Fritsche LG, Igl W, Bailey JN, Grassmann F, Sengupta S, Bragg-Gresham JL, Burdon KP, Hebbring SJ, Wen C, Gorski M, Kim IK, Cho D, Zack D, Souied E, Scholl HP, Bala E, Lee KE, Hunter DJ, Sardell RJ, Mitchell P, Merriam JE, Cipriani V, Hoffman JD, Schick T, Lechanteur YT, Guymer RH, Johnson MP, Jiang Y, Stanton CM, Buitendijk GH, Zhan X, Kwong AM, Boleda A, Brooks M, Gieser L, Ratnapriya R, Branham KE, Foerster JR, Heckenlively JR, Othman MI, Vote BJ, Liang HH, Souzeau E, McAllister IL, Isaacs T, Hall J, Lake S, Mackey DA, Constable IJ, Craig JE, Kitchner TE, Yang Z, Su Z, Luo H, Chen D, Ouyang H, Flagg K, Lin D, Mao G, Ferreyra H, Stark K, von Strachwitz CN, Wolf A, Brandl C, Rudolph G, Olden M, Morrison MA, Morgan DJ, Schu M, Ahn J, Silvestri G, Tsironi EE, Park KH, Farrer LA, Orlin A, Brucker A, Li M, Curcio CA, Mohand-Said S, Sahel JA, Audo I, Benchaboune M, Cree AJ, Rennie CA, Goverdhan SV, Grunin M, Hagbi-Levi S, Campochiaro P, Katsanis N, Holz FG, Blond F, Blanché H, Deleuze JF, Igo RP, Truitt B, Peachey NS, Meuer SM, Myers CE, Moore EL, Klein R, Hauser MA, Postel EA, Courtenay MD, Schwartz SG, Kovach JL, Scott WK, Liew G, Tan AG, Gopinath B, Merriam JC, Smith RT, Khan JC, Shahid H, Moore AT, McGrath JA, Laux R, Brantley MA, Agarwal A, Ersoy L, Caramoy A, Langmann T, Saksens NT, de Jong EK, Hoyng CB, Cain MS, Richardson AJ, Martin TM, Blangero J, Weeks DE, Dhillon B, van Duijn CM, Doheny KF, Romm J, Klaver CC, Hayward C, Gorin MB, Klein ML, Baird PN, den Hollander AI, Fauser S, Yates JR, Allikmets R, Wang JJ, Schaumberg DA, Klein BE, Hagstrom SA, Chowers I, Lotery AJ, Léveillard T, Zhang K, Brilliant MH, Hewitt AW, Swaroop A, Chew EY, Pericak-Vance MA, DeAngelis M, Stambolian D, Haines JL, Iyengar SK, Weber BH, Abecasis GR, Heid IM (2016). A large genome-wide association study of age-related macular degeneration highlights contributions of rare and common variants. Nat Genet.

[27] Schramm EC, Clark SJ, Triebwasser MP, Raychaudhuri S, Seddon JM, Atkinson JP (2014). Genetic variants in the complement system predisposing to age-related macular degeneration: a review. Mol Immunol.

[28] Bhisitkul RB (2006). Vascular endothelial growth factor biology: clinical implications for ocular treatments. Br J Ophthalmol.

[29] Constable IJ, Lai CM, Magno AL, French MA, Barone SB, Schwartz SD, Blumenkranz MS, Degli-Esposti MA, Rakoczy EP (2017). Gene therapy in neovascular age-related macular degeneration: three-year follow-up of a phase 1 randomized dose escalation trial. Am J Ophthalmol.

[30] Heier JS, Kherani S, Desai S, Dugel P, Kaushal S, Cheng SH, Delacono C, Purvis A, Richards S, Le-Halpere A, Connelly J, Wadsworth SC, Varona R, Buggage R, Scaria A, Campochiaro PA (2017). Intravitreous injection of AAV2-sFLT01 in patients with advanced neovascular age-related macular degeneration: a phase 1, open-label trial. Lancet.

[31] Bloquel C, Bourges JL, Touchard E, Berdugo M, BenEzra D, Behar-Cohen F (2006). Non-viral ocular gene therapy: potential ocular therapeutic avenues. Adv Drug Deliv Rev.

[32] Cheng HC, Yeh SI, Tsao YP, Kuo PC (2007). Subconjunctival injection of recombinant AAV-angiostatin ameliorates alkali burn induced corneal angiogenesis. Mol Vis.

[33] Yoon KC, Bae JA, Park HJ, Im SK, Oh HJ, Lin XH, Kim MY, Lee JH, Lee SE, Ahn KY, Kim KK (2009). Subconjunctival gene delivery of the transcription factor GA-binding protein delays corneal neovascularization in a mouse model. Gene Ther.

[34] Gerometta R, Spiga MG, Borras T, Candia OA (2010). Treatment of sheep steroid-induced ocular hypertension with a glucocorticoid-inducible MMP1 gene therapy virus. Invest Ophthalmol Vis Sci.

[35] Dalkara D, Kolstad KD, Caporale N, Visel M, Klimczak RR, Schaffer DV, Flannery JG (2009). Inner limiting membrane barriers to AAV-mediated retinal transduction from the vitreous. Mol Ther.

[36] Takahashi K, Igarashi T, Miyake K, Kobayashi M, Yaguchi C, Iijima O, Yamazaki Y, Katakai Y, Miyake N, Kameya S, Shimada T, Takahashi H, Okada T (2017). Improved intravitreal AAV-mediated inner retinal gene transduction after surgical internal limiting membrane peeling in cynomolgus monkeys. Mol Ther.

[37] Bruewer AR, Mowat FM, Bartoe JT, Boye SL, Hauswirth WW, Petersen-Jones SM (2013). Evaluation of lateral spread of transgene expression following subretinal AAV-mediated gene delivery in dogs. PLoS One.

[38] Peden MC, Min J, Meyers C, Lukowski Z, Li Q, Boye SL, Levine M, Hauswirth WW, Ratnakaram R, Dawson W, Smith WC, Sherwood MB (2011). Ab-externo AAV-mediated gene delivery to the suprachoroidal space using a 250 micron flexible microcatheter. PLoS One.

[39] Ramamoorth M, Narvekar A (2015). Non viral vectors in gene therapy- an overview. J Clin Diagn Res.

[40] Kachi S, Oshima Y, Esumi N, Kachi M, Rogers B, Zack DJ, Campochiaro PA (2005). Nonviral ocular gene transfer. Gene Ther.

[41] Delgado D, del Pozo-Rodriguez A, Solinís MÁ, Avilés-Triqueros M, Weber BH, Fernández E, Gascón AR (2012). Dextran and protamine-based solid lipid nanoparticles as potential vectors for the treatment of X-linked juvenile retinoschisis. Hum Gene Ther.

[42] Pitkanen L, Ruponen M, Nieminen J, Urtti A (2003). Vitreous is a barrier in nonviral gene transfer by cationic lipids and polymers. Pharm Res.

[43] Bejjani RA, BenEzra D, Cohen H, Rieger J, Andrieu C, Jeanny JC, Gollomb G, Behar-Cohen FF (2005). Nanoparticles for gene delivery to retinal pigment epithelial cells. Mol Vis.

[44] Bourges JL, Gautier SE, Delie F, Bejjani RA, Jeanny JC, Gurny R, BenEzra D, Behar-Cohen FF (2003). Ocular drug delivery targeting the retina and retinal pigment epithelium using polylactide nanoparticles. Invest Ophthalmol Vis Sci.

[45] Trapani I, Puppo A, Auricchio A (2014). Vector platforms for gene therapy of inherited retinopathies. Prog Retin Eye Res.

[46] Kong J, Kim SR, Binley K, Pata I, Doi K, Mannik J, Zernant-Rajang J, Kan O, Iqball S, Naylor S, Sparrow JR, Gouras P, Allikmets R (2008). Correction of the disease phenotype in the mouse model of Stargardt disease by lentiviral gene therapy. Gene Ther.

[47] Auricchio A, Kobinger G, Anand V, Hildinger M, O'Connor E, Maguire AM, Wilson JM, Bennett J (2001). Exchange of surface proteins impacts on viral vector cellular specificity and transduction characteristics: the retina as a model. Hum Mol Genet.

[48] Zufferey R, Dull T, Mandel RJ, Bukovsky A, Quiroz D, Naldini L, Trono D (1998). Self-inactivating lentivirus vector for safe and efficient in vivo gene delivery. J Virol.

[49] Yáñez-Muñoz RJ, Balaggan KS, MacNeil A, Howe SJ, Schmidt M, Smith AJ, Buch P, MacLaren RE, Anderson PN, Barker SE, Duran Y, Bartholomae C, von Kalle C, Heckenlively JR, Kinnon C, Ali RR, Thrasher AJ (2006). Effective gene therapy with nonintegrating lentiviral vectors. Nat Med.

[50] Chen Y, Moiseyev G, Takahashi Y, Ma JX (2006). RPE65 gene delivery restores isomerohydrolase activity and prevents early cone loss in *Rpe65*^−^/^−^ mice. Invest Ophthalmol Vis Sci.

[51] Vollrath D, Feng W, Duncan JL, Yasumura D, D'Cruz PM, Chappelow A, Matthes MT, Kay MA, LaVail MM (2001). Correction of the retinal dystrophy phenotype of the RCS rat by viral gene transfer of *Mertk*. Proc Natl Acad Sci U S A.

[52] Campochiaro PA, Nguyen QD, Shah SM, Klein ML, Holz E, Frank RN, Saperstein DA, Gupta A, Stout JT, Macko J, DiBartolomeo R, Wei LL (2006). Adenoviral vector-delivered pigment epithelium-derived factor for neovascular age-related macular degeneration: results of a phase I clinical trial. Hum Gene Ther.

[53] Reichel MB, Ali RR, Thrasher AJ, Hunt DM, Bhattacharya SS, Baker D (1998). Immune responses limit adenovirally mediated gene expression in the adult mouse eye. Gene Ther.

[54] Parks RJ, Chen L, Anton M, Sankar U, Rudnicki MA, Graham FL (1996). A helper-dependent adenovirus vector system: removal of helper virus by Cre-mediated excision of the viral packaging signal. Proc Natl Acad Sci U S A.

[55] Schön C, Biel M, Michalakis S (2015). Retinal gene delivery by adeno-associated virus (AAV) vectors: strategies and applications. Eur J Pharm Biopharm.

[56] Lotery AJ, Yang GS, Mullins RF, Russell SR, Schmidt M, Stone EM, Lindbloom JD, Chiorini JA, Kotin RM, Davidson BL (2003). Adeno-associated virus type 5: transduction efficiency and cell-type specificity in the primate retina. Hum Gene Ther.

[57] Yang GS, Schmidt M, Yan Z, Lindbloom JD, Harding TC, Donahue BA, Engelhardt JF, Kotin R, Davidson BL (2002). Virus-mediated transduction of murine retina with adeno-associated virus: effects of viral capsid and genome size. J Virol.

[58] Weber M, Rabinowitz J, Provost N, Conrath H, Folliot S, Briot D, Chérel Y, Chenuaud P, Samulski J, Moullier P, Rolling F (2003). Recombinant adeno-associated virus serotype 4 mediates unique and exclusive long-term transduction of retinal pigmented epithelium in rat, dog, and nonhuman primate after subretinal delivery. Mol Ther.

[59] Madigan VJ, Asokan A (2016). Engineering AAV receptor footprints for gene therapy. Curr Opin Virol.

[60] Gao G, Vandenberghe LH, Wilson JM (2005). New recombinant serotypes of AAV vectors. Curr Gene Ther.

[61] Vandenberghe LH, Bell P, Maguire AM, Cearley CN, Xiao R, Calcedo R, Wang L, Castle MJ, Maguire AC, Grant R, Wolfe JH, Wilson JM, Bennett J (2011). Dosage thresholds for AAV2 and AAV8 photoreceptor gene therapy in monkey. Sci Transl Med.

[62] Auricchio A (2003). Pseudotyped AAV vectors for constitutive and regulated gene expression in the eye. Vis Res.

[63] Calcedo R, Vandenberghe LH, Gao G, Lin J, Wilson JM (2009). Worldwide epidemiology of neutralizing antibodies to adeno-associated viruses. J Infect Dis.

[64] Allocca M, Mussolino C, Garcia-Hoyos M, Sanges D, Iodice C, Petrillo M, Vandenberghe LH, Wilson JM, Marigo V, Surace EM, Auricchio A (2007). Novel adeno-associated virus serotypes efficiently transduce murine photoreceptors. J Virol.

[65] Lebherz C, Maguire A, Tang W, Bennett J, Wilson JM (2008). Novel AAV serotypes for improved ocular gene transfer. J Gene Med.

[66] Manfredi A, Marrocco E, Puppo A, Cesi G, Sommella A, Della Corte M, Rossi S, Giunti M, Craft CM, Bacci ML, Simonelli F, Surace EM, Auricchio A (2013). Combined rod and cone transduction by adeno-associated virus 2/8. Hum Gene Ther.

[67] Mussolino C, della Corte M, Rossi S, Viola F, Di Vicino U, Marrocco E, Neglia S, Doria M, Testa F, Giovannoni R, Crasta M, Giunti M, Villani E, Lavitrano M, Bacci ML, Ratiglia R, Simonelli F, Auricchio A, Surace EM (2011). AAV-mediated photoreceptor transduction of the pig cone-enriched retina. Gene Ther.

[68] Alexander JJ, Umino Y, Everhart D, Chang B, Min SH, Li Q, Timmers AM, Hawes NL, Pang JJ, Barlow RB, Hauswirth WW (2007). Restoration of cone vision in a mouse model of achromatopsia. Nat Med.

[69] Komaromy AM, Alexander JJ, Rowlan JS, Garcia MM, Chiodo VA, Kaya A, Tanaka JC, Acland GM, Hauswirth WW, Aguirre GD (2010). Gene therapy rescues cone function in congenital achromatopsia. Hum Mol Genet.

[70] Mancuso K, Hauswirth WW, Li Q, Connor TB, Kuchenbecker JA, Mauck MC, Neitz J, Neitz M (2009). Gene therapy for red-green colour blindness in adult primates. Nature.

[71] Kotterman MA, Schaffer DV (2014). Engineering adeno-associated viruses for clinical gene therapy. Nat Rev Genet.

[72] Zhong L, Li B, Mah CS, Govindasamy L, Agbandje-McKenna M, Cooper M, Herzog RW, Zolotukhin I, Warrington KH, Weigel-Van Aken KA, Hobbs JA, Zolotukhin S, Muzyczka N, Srivastava A (2008). Next generation of adeno-associated virus 2 vectors: point mutations in tyrosines lead to high-efficiency transduction at lower doses. Proc Natl Acad Sci U S A.

[73] Petrs-Silva H, Dinculescu A, Li Q, Min SH, Chiodo V, Pang JJ, Zhong L, Zolotukhin S, Srivastava A, Lewin AS, Hauswirth WW (2009). High-efficiency transduction of the mouse retina by tyrosine-mutant AAV serotype vectors. Mol Ther.

[74] Martino AT, Basner-Tschakarjan E, Markusic DM, Finn JD, Hinderer C, Zhou S, Ostrov DA, Srivastava A, Ertl HC, Terhorst C, High KA, Mingozzi F, Herzog RW (2013). Engineered AAV vector minimizes in vivo targeting of transduced hepatocytes by capsid-specific CD8^+^ T cells. Blood.

[75] Dalkara D, Byrne LC, Klimczak RR, Visel M, Yin L, Merigan WH, Flannery JG, Schaffer DV. In vivo–directed evolution of a new adeno-associated virus for therapeutic outer retinal gene delivery from the vitreous. Sci Transl Med 2013;5(189):189ra176. 10.1126/scitranslmed.3005708 23761039

[76] Dalkara D, Kolstad KD, Guerin KI, Hoffmann NV, Visel M, Klimczak RR, Schaffer DV, Flannery JG (2011). AAV mediated GDNF secretion from retinal glia slows down retinal degeneration in a rat model of retinitis pigmentosa. Mol Ther.

[77] Klimczak RR, Koerber JT, Dalkara D, Flannery JG, Schaffer DV (2009). A novel adeno-associated viral variant for efficient and selective intravitreal transduction of rat Muller cells. PLoS One.

[78] Young JE, Vogt T, Gross KW, Khani SC (2003). A short, highly active photoreceptor-specific enhancer/promoter region upstream of the human rhodopsin kinase gene. Invest Ophthalmol Vis Sci.

[79] Beltran WA, Cideciyan AV, Lewin AS, Iwabe S, Khanna H, Sumaroka A, Chiodo VA, Fajardo DS, Román AJ, Deng WT, Swider M, Alemán TS, Boye SL, Genini S, Swaroop A, Hauswirth WW, Jacobson SG, Aguirre GD (2012). Gene therapy rescues photoreceptor blindness in dogs and paves the way for treating human X-linked retinitis pigmentosa. Proc Natl Acad Sci U S A.

[80] Flannery JG, Zolotukhin S, Vaquero MI, LaVail MM, Muzyczka N, Hauswirth WW (1997). Efficient photoreceptor-targeted gene expression in vivo by recombinant adeno-associated virus. Proc Natl Acad Sci U S A.

[81] Carvalho LS, Xu J, Pearson RA, Smith AJ, Bainbridge JW, Morris LM, Fliesler SJ, Ding XQ, Ali RR (2011). Long-term and age-dependent restoration of visual function in a mouse model of CNGB3-associated achromatopsia following gene therapy. Hum Mol Genet.

[82] Michalakis S, Mühlfriedel R, Tanimoto N, Krishnamoorthy V, Koch S, Fischer MD, Becirovic E, Bai L, Huber G, Beck SC, Fahl E, Büning H, Paquet-Durand F, Zong X, Gollisch T, Biel M, Seeliger MW (2010). Restoration of cone vision in the CNGA3^−/−^ mouse model of congenital complete lack of cone photoreceptor function. Mol Ther.

[83] Komáromy AM, Alexander JJ, Cooper AE, Chiodo VA, Glushakova LG, Acland GM, Hauswirth WW, Aguirre GD (2008). Targeting gene expression to cones with human cone opsin promoters in recombinant AAV. Gene Ther.

[84] Nicoletti A, Kawase K, Thompson DA (1998). Promoter analysis of RPE65, the gene encoding a 61-kDa retinal pigment epithelium-specific protein. Invest Ophthalmol Vis Sci.

[85] Esumi N, Oshima Y, Li Y, Campochiaro PA, Zack DJ (2004). Analysis of the *VMD2* promoter and implication of E-box binding factors in its regulation. J Biol Chem.

[86] Doroudchi MM, Greenberg KP, Liu J, Silka KA, Boyden ES, Lockridge JA, Arman AC, Janani R, Boye SE, Boye SL, Gordon GM, Matteo BC, Sampath AP, Hauswirth WW, Horsager A (2011). Virally delivered channelrhodopsin-2 safely and effectively restores visual function in multiple mouse models of blindness. Mol Ther.

[87] Macé E, Caplette R, Marre O, Sengupta A, Chaffiol A, Barbe P, Desrosiers M, Bamberg E, Sahel JA, Picaud S, Duebel J, Dalkara D (2015). Targeting channelrhodopsin-2 to ON-bipolar cells with vitreally administered AAV restores ON and OFF visual responses in blind mice. Mol Ther.

[88] Aartsen WM, van Cleef KW, Pellissier LP, Hoek RM, Vos RM, Blits B, Ehlert EM, Balaggan KS, Ali RR, Verhaagen J, Wijnholds J (2010). GFAP-driven GFP expression in activated mouse Muller glial cells aligning retinal blood vessels following intravitreal injection of AAV2/6 vectors. PLoS One.

[89] Ayuso E, Mingozzi F, Bosch F (2010). Production, purification and characterization of adeno-associated vectors. Curr Gene Ther..

[90] Wright JF (2014). Product-related impurities in clinical-grade recombinant AAV vectors: characterization and risk assessment. Biomedicines.

[91] Wright JF, Wellman J, High KA (2010). Manufacturing and regulatory strategies for clinical AAV2-hRPE65. Curr Gene Ther..

[92] Schnodt M, Büning H (2017). Improving the quality of adeno-associated viral vector preparations: the challenge of product-related impurities. Hum Gene Ther Methods.

[93] Wright JF, Le T, Prado J, Bahr-Davidson J, Smith PH, Zhen Z, Sommer JM, Pierce GF, Qu G (2005). Identification of factors that contribute to recombinant AAV2 particle aggregation and methods to prevent its occurrence during vector purification and formulation. Mol Ther.

[94] Clément N, Grieger JC (2016). Manufacturing of recombinant adeno-associated viral vectors for clinical trials. Mol Ther Methods Clin Dev..

[95] Summerford C, Samulski RJ (1999). Viral receptors and vector purification: new approaches for generating clinical-grade reagents. Nat Med.

[96] Nass SA, Mattingly MA, Woodcock DA, Burnham BL, Ardinger JA, Osmond SE, Frederick AM, Scaria A, Cheng SH, O'Riordan CR (2018). Universal method for the purification of recombinant AAV vectors of differing serotypes. Mol Ther Methods Clin Dev.

[97] Qu G, Bahr-Davidson J, Prado J, Tai A, Cataniag F, McDonnell J, Zhou J, Hauck B, Luna J, Sommer JM, Smith P, Zhou S, Colosi P, High KA, Pierce GF, Wright JF (2007). Separation of adeno-associated virus type 2 empty particles from genome containing vectors by anion-exchange column chromatography. J Virol Methods.

[98] Shalaev EY, Wang W, Gatlin LA, McNally EJ, Hastedt JE (2008). Rational choice of excipients of use in lyophilized formulations. Protein formulation and delivery.

[99] Singh S, Kolhe P, Wang W, Nema S. Large-scale freezing of biologics. Bioprocess Int. 2009;7(9):32–44.

[100] Wu C, Shamblin S, Varshney D, Shalaev E, Varshney D, Singh M (2015). Advance understanding of buffer behavior during lyophilization. Lyophilized biologics and vaccines: modality-based approaches.

[101] Croyle MA, Cheng X, Wilson JM (2001). Development of formulations that enhance physical stability of viral vectors for gene therapy. Gene Ther.

[102] Venkatakrishnan B, Yarbrough J, Domsic J, Bennett A, Bothner B, Kozyreva OG, Samulski RJ, Muzyczka N, McKenna R, Agbandje-McKenna M (2013). Structure and dynamics of adeno-associated virus serotype 1 VP1-unique N-terminal domain and its role in capsid trafficking. J Virol.

[103] Xie Q, Hare J, Turnigan J, Chapman MS (2004). Large-scale production, purification and crystallization of wild-type adeno-associated virus-2. J Virol Methods.

[104] Wang P, Li H, Yang HJ, Wang HB, Lu JH, Zhang Y, Hu J (2007). Glycerol facilitates the disaggregation of recombinant adeno-associated virus serotype 2 on mica surface. Colloids Surf B: Biointerfaces.

[105] Bennicelli J, Wright JF, Komaromy A, Jacobs JB, Hauck B, Zelenaia O, Mingozzi F, Hui D, Chung D, Rex TS, Wei Z, Qu G, Zhou S, Zeiss C, Arruda VR, Acland GM, Dell'Osso LF, High KA, Maguire AM, Bennett J (2008). Reversal of blindness in animal models of leber congenital amaurosis using optimized AAV2-mediated gene transfer. Mol Ther.

[106] Sommer J, Sanftner L, Feng L, Suzuki B, Zhen Z, Powell S, Wright F, McClelland A, Cunningham J (2002). Optimal recovery of AAV-AADC vector from Teflon catheters using Polysorbate 80 or Pluronic F68 (abstract 768). Mol Ther.

[107] Shalaev E, Franks F. Solid-liquid state diagrams in pharmaceutical lyophilisation: crystallization of solutes. In: Levine H, editor. Amorphous food and pharmaceutical systems. London: The Royal Society of Chemistry; 2002. p. 200–15.

[108] Anzo K, Harada M, Okada T (2013). Enhanced kinetics of pseudo first-order hydrolysis in liquid phase coexistent with ice. J Phys Chem A.

[109] Bhatnagar BS, Bogner RH, Pikal MJ (2007). Protein stability during freezing: separation of stresses and mechanisms of protein stabilization. Pharm Dev Technol.

[110] Bhatnagar BS, Pikal MJ, Bogner RH (2008). Study of the individual contributions of ice formation and freeze-concentration on isothermal stability of lactate dehydrogenase during freezing. J Pharm Sci.

[111] Franks F, Hatley RHM (1991). Stability of proteins at subzero temperatures: thermodynamics and some ecological consequences. Pure Appl Chem.

[112] Lund DB, Fennema OR, Powrie WD (1969). Enzymic and acid hydrolysis of sucrose as influenced by freezing. J Food Sci.

[113] Pincock RE (1969). Reactions in frozen systems. Acc Chem Res.

[114] Pincock RE, Kiovsky TE (1966). Kinetics of reactions in frozen solutions. J Chem Educ.

[115] Schwegman JJ, Carpenter JF, Nail SL (2009). Evidence of partial unfolding of proteins at the ice/freeze-concentrate interface by infrared microscopy. J Pharm Sci.

[116] Singh SK, Kolhe P, Mehta AP, Chico SC, Lary AL, Huang M (2011). Frozen state storage instability of a monoclonal antibody: aggregation as a consequence of trehalose crystallization and protein unfolding. Pharm Res.

[117] Wright JF, Qu G, Tang C, Sommer JM (2003). Recombinant adeno-associated virus: formulation challenges and strategies for a gene therapy vector. Curr Opin Drug Discov Devel.

[118] Howard DB, Harvey BK (2017). Assaying the stability and inactivation of AAV serotype 1 vectors. Hum Gene Ther Methods.

[119] Guidance for FDA Reviewers and Sponsors, Content and Review of Chemistry, Manufacturing and Control (CMC) Information for Human Gene Therapy Investigational New Drug Applications (INDs). Food and Drug Administration, Center for Biologics Evaluation and Research. 2008. Available from: https://www.fda.gov/downloads/biologicsbloodvaccines/guidancecomplianceregulatoryinformation/guidances/cellularandgenetherapy/ucm078694.pdf. Accessed 20 Nov 2018.

[120] D'Costa S, Blouin V, Broucque F, Penaud-Budloo M, François A, Perez IC, Le Bec C, Moullier P, Snyder RO, Ayuso E (2016). Practical utilization of recombinant AAV vector reference standards: focus on vector genomes titration by free ITR qPCR. Mol Ther Methods Clin Dev.

[121] Neuberger EW, Perez I, Le Guiner C, Moser D, Ehlert T, Allais M, Moullier P, Simon P, Snyder RO (2016). Establishment of two quantitative nested qPCR assays targeting the human EPO transgene. Gene Ther.

[122] Sommer JM, Smith PH, Parthasarathy S, Isaacs J, Vijay S, Kieran J, Powell SK, McClelland A, Wright JF (2003). Quantification of adeno-associated virus particles and empty capsids by optical density measurement. Mol Ther.

[123] Joo KI, Fang Y, Liu Y, Xiao L, Gu Z, Tai A, Lee CL, Tang Y, Wang P (2011). Enhanced real-time monitoring of adeno-associated virus trafficking by virus-quantum dot conjugates. ACS Nano.

[124] Bennett A, Patel S, Mietzsch M, Jose A, Lins-Austin B, Yu JC, Bothner B, McKenna R, Agbandje-McKenna M (2017). Thermal stability as a determinant of AAV serotype identity. Mol Ther Methods Clin Dev.

[125] U.S. Food and Drug Administration Center for Biologics Evaluation and Research. Chemistry, manufacturing, and control (CMC) information for human gene therapy investigational new drug applications (INDs): draft guidance for industry. 2018. Available from: https://www.fda.gov/downloads/BiologicsBloodVaccines/GuidanceComplianceRegulatoryInformation/Guidances/CellularandGeneTherapy/UCM610795.pdf. Accessed 20 Nov 2018.

[126] U.S. Food and Drug Administration Center for Biologics Evaluation and Research. Human gene therapy for retinal disorders: draft guidance for industry. 2018. Available from: https://www.fda.gov/downloads/BiologicsBloodVaccines/GuidanceComplianceRegulatoryInformation/Guidances/CellularandGeneTherapy/UCM610803.pdf. Accessed 20 Nov 2018.

[127] U.S. Food and Drug Administration Center for Biologics Evaluation and Research. Testing of retroviral vector-based human gene therapy products for replication competent retrovirus during product manufacture and patient follow-up: draft guidance for industry. 2018. Available from: https://www.fda.gov/downloads/BiologicsBloodVaccines/GuidanceComplianceRegulatoryInformation/Guidances/CellularandGeneTherapy/UCM610800.pdf. Accessed 20 Nov 2018.

[128] U.S. Food and Drug Administration Center for Biologics Evaluation and Research. Human gene therapy for hemophilia: draft guidance for industry. 2018. Available from: https://www.fda.gov/downloads/BiologicsBloodVaccines/GuidanceComplianceRegulatoryInformation/Guidances/CellularandGeneTherapy/UCM610801.pdf. Accessed 20 Nov 2018.

[129] U.S. Food and Drug Administration Center for Biologics Evaluation and Research. Human gene therapy for rare diseases: draft guidance for industry. 2018. Available from: https://www.fda.gov/downloads/BiologicsBloodVaccines/GuidanceComplianceRegulatoryInformation/Guidances/CellularandGeneTherapy/UCM610802.pdf. Accessed 20 Nov 2018.

[130] U.S. Food and Drug Administration Center for Biologics Evaluation and Research. Long term follow-up after administration of human gene therapy products: draft guidance for industry. 2018. Available from: https://www.fda.gov/downloads/BiologicsBloodVaccines/GuidanceComplianceRegulatoryInformation/Guidances/CellularandGeneTherapy/UCM610797.pdf. Accessed 20 Nov 2018.

[131] European Medicines Agency. Guideline on the quality, non-clinical and clinical aspects of gene therapy medicinal products. 2018. Available from: http://www.ema.europa.eu/docs/en_GB/document_library/Scientific_guideline/2018/07/WC500252056.pdf. Accessed 20 Nov 2018.

[132] U.S. Food and Drug Administration Center for Biologics Evaluation and Research. Design and analysis of shedding studies for virus or bacteria-based gene therapy and oncolytic products: guidance for industry. 2015. Available from: https://www.fda.gov/downloads/Guidances/UCM404087.pdf. Accessed 20 Nov 2018.

[133] European Medicines Agency. ICH considerations -- general principles to address virus and vector shedding. 2009. Available from: http://www.ema.europa.eu/docs/en_GB/document_library/Scientific_guideline/2009/09/WC500002680.pdf. Accessed 20 Nov 2018.

[134] International Council for Harmonisation of Technical Requirements for Pharmaceuticals for Human Use (ICH). Guideline on virus and gene therapy vector shedding and transmission. 2009. Available from: http://www.ich.org/products/guidelines/multidisciplinary/multidisciplinary-single/article/virus-and-gene-therapy-vector-shedding-and-transmission.html. Accessed 20 Nov 2018.

[135] U.S. Food and Drug Administration. Summary basis for regulatory action -- LUXTURNA. Available from: https://www.fda.gov/downloads/BiologicsBloodVaccines/CellularGeneTherapyProducts/ApprovedProducts/UCM592083.pdf. Accessed 20 Nov 2018.

